# Surface Electron-Hole Rich Species Active in the Electrocatalytic Water Oxidation

**DOI:** 10.1021/jacs.1c01655

**Published:** 2021-08-06

**Authors:** Juan-Jesús Velasco-Vélez, Emilia A. Carbonio, Cheng-Hao Chuang, Cheng-Jhih Hsu, Jyh-Fu Lee, Rosa Arrigo, Michael Hävecker, Ruizhi Wang, Milivoj Plodinec, Feng Ryan Wang, Alba Centeno, Amaia Zurutuza, Lorenz J. Falling, Rik Valentijn Mom, Stephan Hofmann, Robert Schlögl, Axel Knop-Gericke, Travis E. Jones

**Affiliations:** † Department of Heterogeneous Reactions, Max Planck Institute for Chemical Energy Conversion, Mülheim an der Ruhr 45470, Germany; ‡ Department of Inorganic Chemistry, Fritz-Haber-Institut der Max-Planck-Gesellschaft, Berlin 14195, Germany; § Helmholtz-Center Berlin for Materials and Energy, BESSY II, Berlin 12489, Germany; ∥ Department of Physics, Tamkang University, New Taipei City 25137, Taiwan; ⊥ National Synchrotron Radiation Research Center, Hsinchu 30076, Taiwan; # School of Sciences, University of Salford, Environment and Life, Cockcroft building, M5 4WT, Manchester, U.K.; ¶ Department of Engineering, University of Cambridge, Cambridge CB3 0FA, U.K.; □ Rudjer Boskovic Institute, Bijenicka 54, HR-10000 Zagreb, Croatia; ○ Department of Chemical Engineering, University College London, Torrington Placa, London WC1E7JE, U.K.; △ Graphenea, San Sebastian 20018, Spain

## Abstract

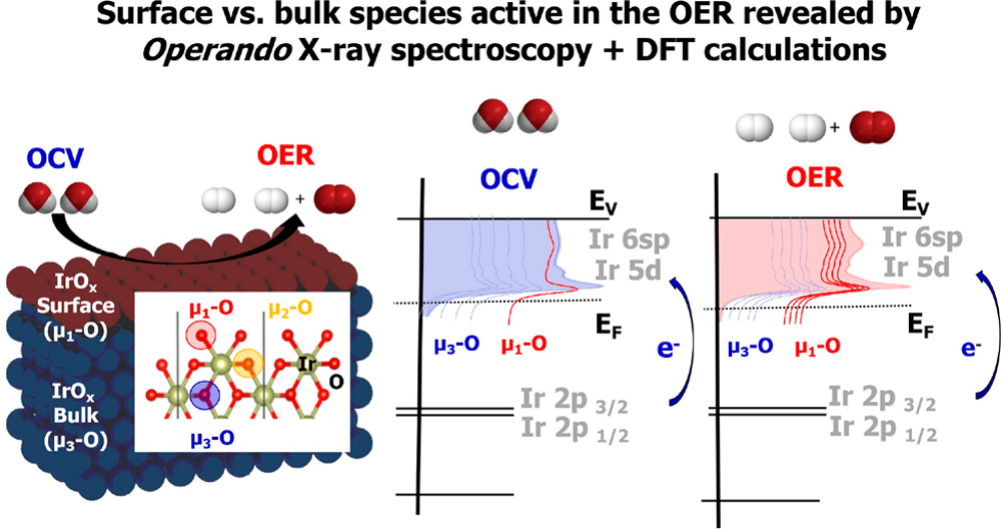

Iridium and ruthenium and their oxides/hydroxides are the best candidates for the oxygen evolution reaction under harsh acidic conditions owing to the low overpotentials observed for Ru- and Ir-based anodes and the high corrosion resistance of Ir-oxides. Herein, by means of cutting edge *operando* surface and bulk sensitive X-ray spectroscopy techniques, specifically designed electrode nanofabrication and *ab initio* DFT calculations, we were able to reveal the electronic structure of the active IrO_*x*_ centers (i.e., oxidation state) during electrocatalytic oxidation of water in the surface and bulk of high-performance Ir-based catalysts. We found the oxygen evolution reaction is controlled by the formation of empty Ir 5d states in the surface ascribed to the formation of formally Ir^V^ species leading to the appearance of electron-deficient oxygen species bound to single iridium atoms (μ_1_-O and μ_1_-OH) that are responsible for water activation and oxidation. Oxygen bound to three iridium centers (μ_3_-O) remains the dominant species in the bulk but do not participate directly in the electrocatalytic reaction, suggesting bulk oxidation is limited. In addition a high coverage of a μ_1_-OO (peroxo) species during the OER is excluded. Moreover, we provide the first photoelectron spectroscopic evidence in bulk electrolyte that the higher surface-to-bulk ratio in thinner electrodes enhances the material usage involving the precipitation of a significant part of the electrode surface and near-surface active species.

## Introduction

Water splitting is among the most important electrochemical processes for the storage of renewable energy, as the molecular hydrogen produced at the cathode can be used as a carbon-free energy vector.^1−3^ However, anode corrosion under acidic conditions and anodic polarization shortens material lifetimes leading to increased costs.^4−6^ Among all the materials investigated for the oxygen evolution reaction (OER), iridium and ruthenium oxides/hydroxides have received considerable attention owing to their low overpotentials; however, only iridium and its oxides/hydroxides are known to combine low overpotential and corrosion resistance under harsh acidic conditions.^7,8^ Unfortunately, iridium is also one of the scarcest elements on earth; thus, synthetic strategies for optimizing its usage are required.^9^ To develop such strategies it is important to understand what drives stability and activity in these materials. The presence of a distribution of surface species with a mixed oxidation state between Ir^III^ and Ir^VI^ are suggested^10−12^ to be critical in the nature of the high activity^13^ shown by this element. We reason that determining the electronic structure characteristics responsible for the peculiar OER performance of IrO_*x*_, as well as quantifying the amount of the material and location of the active species participating in the reaction, is key to optimize its electrochemical performance and usage. Herein, we combine *operando* X-ray absorption spectroscopy (XAS) with *operando* X-ray photoelectron spectroscopy (XPS) to provide complementary information on the occupied and unoccupied states. Though XPS gives intrinsically more surface information than XAS, we show how the surface sensitivity of XAS can be enhanced through precise nanofabrication of nanostructure electrodes. By increasing the surface-to-bulk ratio of the electrodes through nanostructuring, the surface information provided by XAS is enhanced, though this does not make XAS a strictly surface-sensitive method. By combining these measurements with *ab initio* calculations, the nature of the active species and their distribution in the surface and near-surface region during the electrocatalytic oxidation of water was determined. It was found that nanostructuring has a significant influence on the overall XAS whiteline intensity as a consequence of the bulk sensitivity of XAS. The XA spectra contain contributions from the different electrode regions (surface and bulk) including the regions of the electrode that are not in direct contact with the electrolyte or other areas where the contact resistance is significantly different due to charge/mass transport limitations. This limits our ability to use XA spectra of bulky electrodes to develop mechanistic pictures of the OER taking place at the electrode/electrolyte interface. Bulky electrodes can also experience chemical changes within different regions as a consequence of slow kinetic processes such as bulk oxidation, different stoichiometries within the electrode, diffusion, and deficient electrolyte wetting. In order to minimize the impact of these unwanted effects and ascribe the changes observed in the electrode to the variation that the electrode surface undergoes during the reaction conditions, different electrode thicknesses were investigated in a well-defined planar electrode configuration, which avoids the use of inks based on ionomers. These results were validated by their comparison with *operando* soft X-ray XPS in the presence of a bulk aqueous electrolyte with the same type of electrodes and conditions as *operando* XAS. The XPS measurements provided key information related to the near-surface oxidation state that were not quantifiable by the XAS measurements alone. To the best of our knowledge, this is the first time that these experiments were performed with *operando* XAS/XPS consecutively with the same type of samples and under the same aqueous bulk electrolyte.

## Experimental Section

### Electrode Preparation

Details of all preparations are given in the Supporting Information (SI). Briefly, two types of electrode were produced, a thin-film bulk model and a nanoparticulate surface model. The bulk model electrode IrO_*x*_ was directly sputtered on a Si_3_N_4_ membrane and activated/oxidize electrochemically by potential cycling. The surface model electrode was prepared in three steps: (i) a graphene layer was transferred onto a 100 nm thick Si_3_N_4_ holey array membrane with 500 nm diameter holes for XPS or a 100 nm thick Si_3_N_4_ X-ray membrane (without holes) for XAS. For the XPS membrane assembly, this process yields a continuous film of graphene with areas supported by the Si_3_N_4_ membranes and areas which are free-standing over the holes in the Si_3_N_4_ holey array membrane, while for the XAS membrane assembly the graphene is fully supported by the Si_3_N_4_ X-ray membrane. In both cases, the membrane can be used as an electrode in electrochemical applications owing to graphene’s good electrically conductivity and the cell’s leak tightness. Moreover, because the graphene is semitransparent to photoelectrons and photons,^14,15^ the graphene coated holey Si_3_N_4_ membrane can be used for *operando* photoelectron spectroscopy under electrochemical conditions,^16,17^ while the fully supported graphene can be used for total fluorescence yield XAS. In order to increase the stability of the graphene membrane for XPS, and cover possible tears/holes in the graphene, a second layer of graphene was transferred to the electrode assembly. SEM measurements of the graphene on the Si_3_N_4_ grid are shown in Figure S1A and B. These two layers of transferred graphene are referred throughout the text as a bilayered graphene (BLG) membrane. Raman spectroscopy^18−21^ was used to check the quality of the crystallized BLG in order to determine the graphitic character of the free standing graphene samples as shown in the SI. (ii) In a second step the graphene electrode was decorated with Ir nanoparticles (NPs) by sputter coating. The particles cover around 20% of the graphene surface, and they range from 2 to 5 nm, with an average diameter of 2.5 nm. (iii) Finally, the Ir NPs were activated/oxidized electrochemically by potential cycling in N_2_ saturated 100 mM H_2_SO_4_ electrolyte. For the *operando* XAS experiments, the fully supported graphene electrode assembly was decorated with 2.5 nm sputtered Ir NPs. The thin film bulk model electrode was prepared by directly sputtering a 20 nm Ir thin film onto the Si_3_N_4_ membrane (100 nm thick, without holes).

### *In Situ* Experiments

The spectra were collected in two different synchrotron facilities. The *in situ* soft X-ray photoelectron spectra were recorded at the ISISS beamline of BESSY II in Berlin using an ambient pressure X-ray photoelectron spectrometer (APXPS). Ir L_3_-edge spectra were measured in the hard X-ray regime at the beamline BL17C1 of the National Synchrotron Radiation Research Center (NSRRC) in Hsinchu (Taiwan). More details of these beamlines can be found in the Supporting Information.

The Ir L_3_ edge spectra were collected using the *in situ* EC-cell shown in Figure S2A, where the flow of liquid was assured with a peristaltic micro pump. This cell is based on a 100 nm thick Si_3_N_4_ membrane which allows the collection of the X-ray absorption total fluorescence yield signal while remaining leak tight.^22^ The XP spectra were collected using a different cell^23,24^ (see Figure S2B) which is compatible with free-standing graphene membranes. The potential and current collection was done via a potentiostat. More details of these approaches can be found in the Supporting Information.

Note that the electrochemical performance and signal collection, as well as the limits and opportunities that these electrochemical cells offer, were discussed in detail elsewhere.^25^ The transmission properties of the photoelectrons traveling through the lens/analyzer are not influenced by the presence of the Si_3_N_4_/BLG as shown in Figure S.3A. The transmission function of the lens and analyzer is smooth and not suddenly dropping. Thus, the transmission function of the lens/hemispherical analyzer remains unaltered when the membrane is incorporated into the system. Furthermore, it is important to remark that the probed depth depends on the KE of the photoelectrons, which is larger at higher excitation energies. In addition, KEs around 500 eV are affected by an overlap with the O KLL Auger spectra, which makes Ir 4f spectrum collection impossible. Otherwise, the cross section of the target element decays at higher excitation energies. Consequently, according to Figure S3B, the best compromise between these two parameters is achieved with a kinetic energy equal to ∼600 eV. Because of this fact, we set the kinetic energy to 600 eV to perform the XP spectra acquisition.

### Calculations

Full details of the simulations can be found in the SI. Briefly, density functional theory calculations were performed at the PBE level using the Quantum ESPRESSO package^26^ using pseudopotentials from the PSLibrary^27^ with a kinetic energy (charge density) cutoff of 60 Ry (600 Ry). The effect of adding a Hubbard *U* correction was studied using *U* = 1.2 eV, which is consistent with the low value expected for Ir.^28^ For bulk rutile-type IrO_2_ calculations a (12 × 12 × 12) ***k***-point mesh was used with Marzari–Vanderbilt cold smearing using a 0.01 Ry smearing parameter,^29^ surface simulations used nearly equivalent ***k***-point meshes. XAS spectra were computed with a resolvent-based Bethe–Salpeter Equation (BSE) approach^30^ using the wave functions from Quantum ESPRESSO with the core-level BSE solver in the OCEAN package.^31^ XPS spectra were computed using a Hopfield perturbation model.^32^

## Results and Disscusion

**Figure 1:**
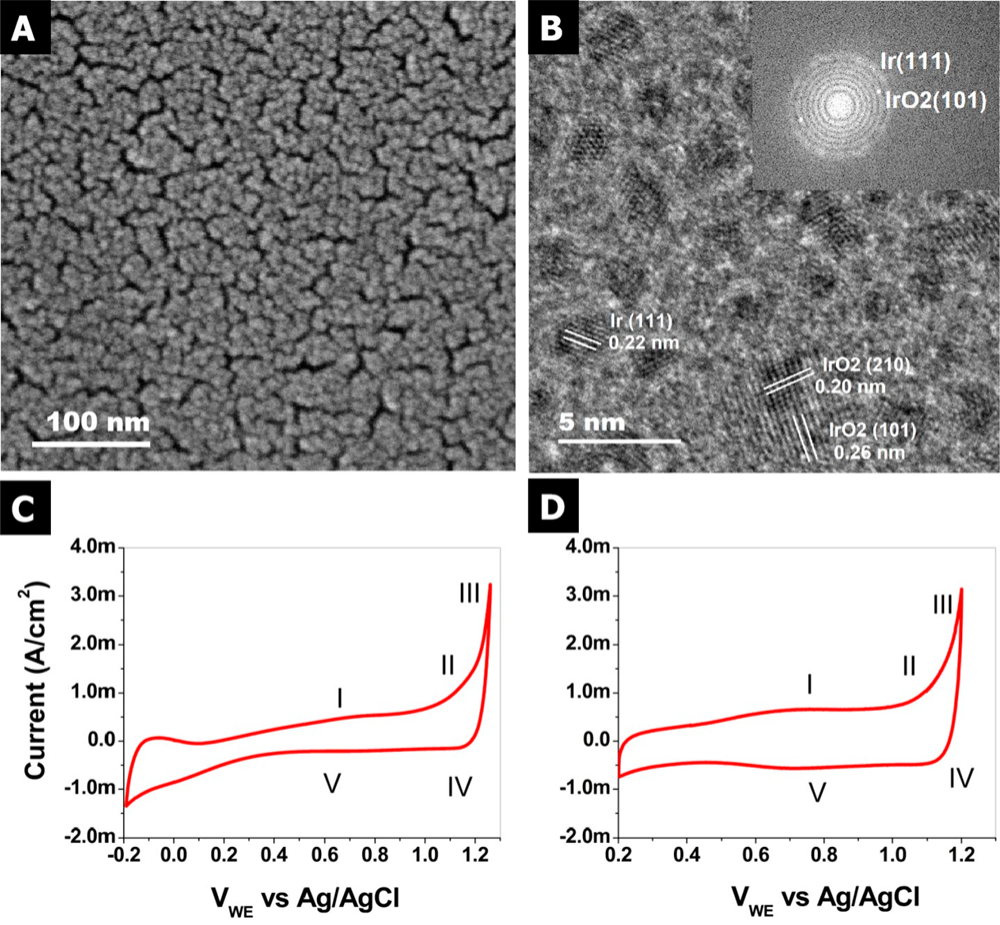
(A) SEM image of the sputtered thin-film IrO_*x*_ electrode. (B) TEM characterization of the sputtered IrO_*x*_ NPs onto the freestanding bilayered graphene (BLG) obtained by CVD. CVs of the (C) thin-film IrOx electrode and (D) IrO_*x*_ NPs in 100 mM H_2_SO_4_ with Pt counter and Ag/AgCl reference electrodes, respectively.

Two different electrodes with different surface-to-bulk ratios were fabricated and compared, namely a 20 nm thin film and 2.5 nm nanoparticles (NPs) sputtered deposited on a graphene current collector electrode. The graphene layer is used as a current collector for the 2.5 nm Ir NPs, where the electrical conductivity between the different Ir NPs is assured by the graphene layer. These two electrodes were characterized with electron microscopy. Thus, Figure 1A shows a top view scanning electron microscopy (SEM) image of the sputtered thin-film electrode, which consists of interconnected polycrystalline islands with a thickness of 20 nm. According to the SEM image in Figure 1A, we estimate a surface area of about 1.5–2.0 qualitative coverage factor (CF) for the thin film and about 1/3 CF for the nanoparticles (with respect to a completely flat surface used as a reference with surface area equal to 1 CF). In contrast, Figure 1B shows the TEM image of the sputtered 2.5 nm IrO_*x*_ NPs supported on a conductive bilayered graphene (BLG) current collector electrode. Owing to the structural differences, the thin film can be considered a bulk model, whereas the high surface-to-bulk ratio of the 2.5 nm Ir NPs is then a surface model. Note that the freshly sputtered electrodes are metallic. It is well-known that the anodic oxidation of metallic iridium is reported to result in better performing catalysts than thermal activation, due to the existence of highly hydrated species forming an oxyhydroxide upon electro-oxidation.^33^ Hence, in order to increase the electrocatalytic performance and ensure the electrode stoichiometry, the sputtered electrodes were oxidized/activated prior to their use during the electrocatalytic oxidation of water to dioxygen (established in this experiments at −1.6 V vs Ag/AgCl) by several potential scans between open circuit voltage (OCV) and 1.2 V at 20 mV/s scanning rate in a 50 mM H_2_SO_4_ electrolyte (potentials are not *iR* corrected). The anodic oxidation results in the development of an increasingly larger anodic peak in the cyclic voltammogram (CV), where the peak height increased with the number of cycles.^34^ The condition of maximum peak height corresponds to the highest electrode activity for thin films and NPs.^35^ Thus, the CV treatment oxidizes Ir metal into the more active IrO_*x*_. After the anodic oxidation, the samples were characterized with the electron microscopy. Thus, the TEM (Figure 1B) images of the sputtered iridium particles on the free-standing bilayer graphene show that they are homogeneously distributed after activation; the estimated coverage factor is around 20% of the surface, and the average particle size is ∼2.5 nm. Contact of the IrO_*x*_ NPs onto graphene is assured by a chemical bond between the surface Ir atoms and the oxygen species present at the edges and vacancies of graphene.^36^ The fast Fourier transform (FFT) proves the existence of reduced metallic iridium NPs (such beam induced reduction is common in nonstoichiometric oxides) and one additional ring ascribed to stoichiometric IrO_2_.

Furthermore, the electrochemical performance of the IrO_*x*_ thin-film and IrO_*x*_ NP electrodes are compared in Figure 1C and 1D. The CVs were acquired at room temperature (25 °C), in deaerated 100 mM H_2_SO_4_ with N_2_ continually bubbled in the electrolyte at a scan rate of 20 mV/s using Pt and Ag/AgCl (saturated in KCl) as counter and reference electrodes, respectively. The CVs show two broad oxidation waves, labeled I and II, and two broad reduction waves, labeled IV and V, due to formally Ir^II^/Ir^IV^ and Ir^IV^/Ir^V^ redox couples (occurring at same potentials), respectively.^34,37^ An additional current (point III in Figure 1C and 1D) is ascribed to the oxidation of water. The CVs in Figure 1C and 1D indicate that both the thin film and bilayer graphene coated with Ir NPs behave similarly. The control experiments with the plain graphene electrode were reported in previous work,^38^ indicating clearly an enhanced catalytic activity for the electrode decorated with Ir NPs. Therefore, it is possible that similar active species are present during the electrocatalytic oxidation of water to dioxygen on both the thin-film and iridium NP electrodes. It is generally accepted that these active sites are hydrated Ir-oxyhydroxides formed *in situ* during OER,^39−41^ where the Ir oxohydroxide-based OER is stable for high-current water electrolysis under acidic conditions.^42^

**Figure 2:**
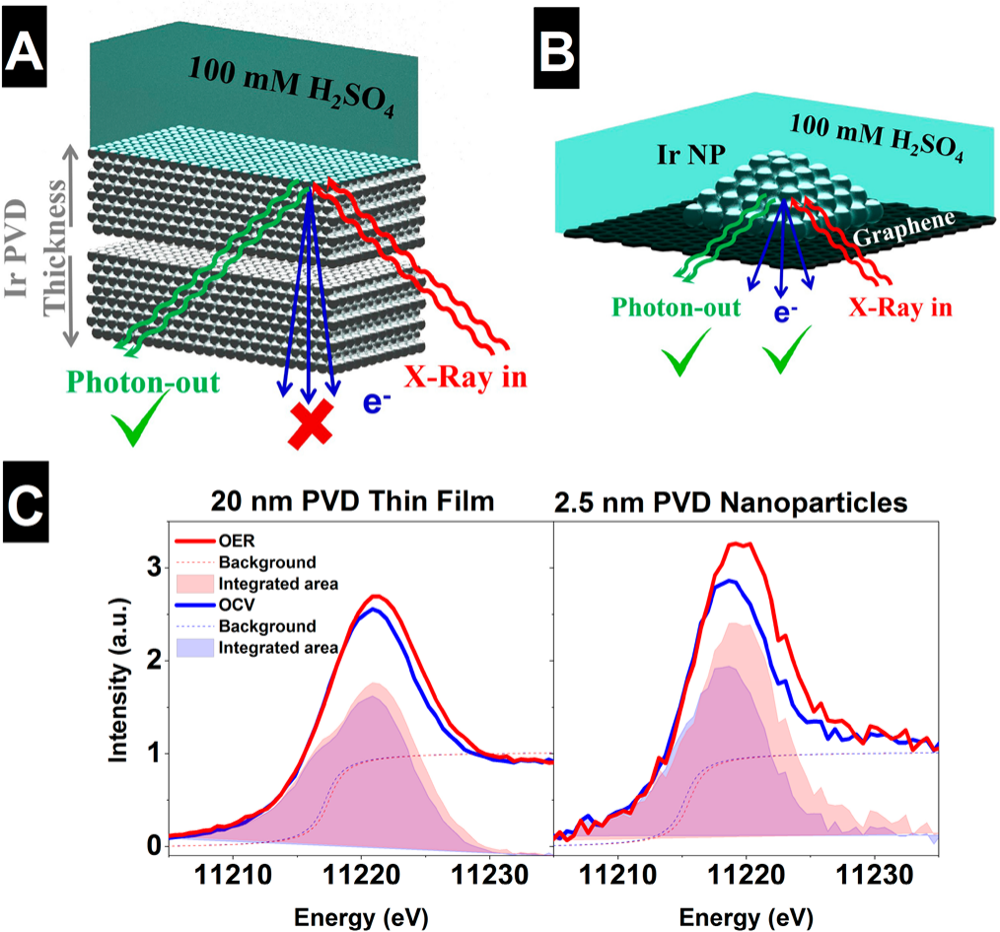
Schematic detection scheme using (A) a thin-film IrO_*x*_ electrode or (B) free-standing CVD bilayer graphene electrode decorated with IrO_*x*_ NPs. Spectra comparison at different potentials (OCV and OER at −1.6 V vs Ag/AgCl) for the (C) thin-film IrO_*x*_ electrode (20 nm) and free-standing CVD bilayered graphene (BLG) electrode decorated with IrO_*x*_ 2.5 nm NPs.

In order to determine the nature of the active species, the electronic structure modification that the electrodes undergo during the electrocatalytic oxidation of water was investigated by means of *operando* XAS. The electrocatalysts were characterized first using XAS in total fluorescence yield (TFY) mode at the Ir L_3_-edge using a homemade *in situ* electrochemical cell;^22^ more details can be found in the Supporting Information (SI). Using a 100 nm thick SiN_*x*_ membrane, which is transparent to the incoming and out-going photons, it is possible to investigate the variations in the electronic structure using photon-in/photon-out (PIPO) techniques in the hard X-ray regime. An advantage of this approach is that it enables the study of electrochemical reactions with aqueous electrolytes (i.e., 100 mM H_2_SO_4_). Figure 2A and 2B show the detection scheme with the thin-film and free-standing bilayer graphene decorated with Ir NPs. For both materials it is possible to perform measurements in TFY-XAS by collecting the photons emitted during the fluorescence decay following the absorption process; this signal comes from the surface and bulk of the materials. Therefore, TFY-XAS provides information associated with the whole electrode (bulk and surface) in the case of the Ir thin film. However, in the case of the NPs, the surface-to-bulk signal is enhanced by a factor of around 10 compared to the thin film electrode, despite the methodology not being a surface informative technique. Moreover, the free-standing graphene allows the collection of photoelectrons (by a hemispherical electron analyzer) from the side exposed to the electrolyte, thereby enabling the acquisition of photoelectron spectra (PES).^43,44^ Meanwhile, the thin-film electrode (20 nm) yields photoelectrons mostly from the side where the electrode is exposed to the incident X-ray photons, as a consequence of the short inelastic mean free path (IMFP) of the low energy photoelectrons accessible within the soft X-ray regime (typically a few nanometers), as shown in Figure 2A. However, using X-rays with higher energy (in the tender or hard X-ray regimen) the information depth can be enlarged at the price of less surface sensitivity. This fact makes it impossible to investigate the electrified solid–liquid interface of such a thick working electrode using soft X-ray PES in this manner. Note that while the short IMFP on soft X-ray PES is a significant technical challenge, the high near-surface sensitivity it offers in solids or liquids^45−47^ makes soft X-ray PES an excellent complement to TFY-XAS.

Figure 2C shows the Ir L_3_-edge spectra collected under *operando* conditions. The Ir L_3_-edge probes the dipole allowed transitions from a core Ir 2p_3/2_ electron to the partially occupied Ir 5d and Ir 6sp orbitals, which are hybridized with the O 2p orbitals.^38^ Transitions to the 5d orbitals are lower in energy and well separated from transitions to the 6sp. These 2p to 5d transitions give rise to the so-called white line. While the large lifetime broadening (about 5 eV^48^) does not allow the discrimination of fine structure in the white line due to, for instance, transitions into t_2g_-like and e_g_-like 5d orbitals, analysis of the white-line intensity can still give insight into the electronic structure of iridium. In particular, a sum rule relates the total number of 5d holes to the integral area of the white line;^11,38^ that is, the white line is linearly proportional to the iridium oxidation state.^48^ Note that while the sum rule is a property of the dipole operator and rigorously holds^49,50^ for L_3_ + L_2_, previous work shows no change in the L_3_/L_2_ branching ratio for oxidized iridium compounds, making L_3_ alone sufficient for a white-line analysis.^48^ The ability of the bulk sensitive TFY-XAS measurements to reveal changes in average Ir oxidation states is apparent from Figure 2C. Using the maximum intensity of the Ir L_3_ edge as a measure of average Ir oxidation state an increase in the number of Ir 5d holes can be seen in both the thin-film and NP samples under anodic polarization. This increase in average oxidation state is reversed under open circuit voltage (OCV). Similar trends were reported previously by *in situ* characterization of iridated working electrodes.^41−55^ Note that the size effects observed in the spectra collected have no clear influence on the catalytic performance of the different electrodes under reaction conditions. We guess that it is a consequence that both planar electrode structures are not very different from one another and consequently the changes in the catalytic performance are not considerable. Before turning to a quantitative evaluation of this behavior, however, the details of the proportionality between white-line intensity and Ir oxidation state must be found. To establish a connection between the white-line intensity and the number of 5d holes on Ir we analyzed a series of reference samples, including: Ir^0^, IrCl_3_, IrO_2_, and IrO_*x*_ (reference samples details can be found in the SI).^54^ An iridium foil was used for Ir^0^. IrCl_3_ powder was used as an Ir^III^ reference, and IrO_2_ rutile-type powder was taken as an Ir^IV^ reference. In addition, an amorphous IrO_*x*_ catalyst rich in active species^40^ was used to compare with the catalysts used in this work, owing to higher electrocatalytic activity compared to the IrO_2_ rutile. Note that the Ir^0^ (foil) white line can be artificially enhanced due to the existence of a native oxide layer, and because this spectrum is collected in TFY mode, self-absorption effects are also possible. However, the oxide layer is not a problem since it is thin compared to the bulk with minor contribution to the white-line intensity.

**Figure 3:**
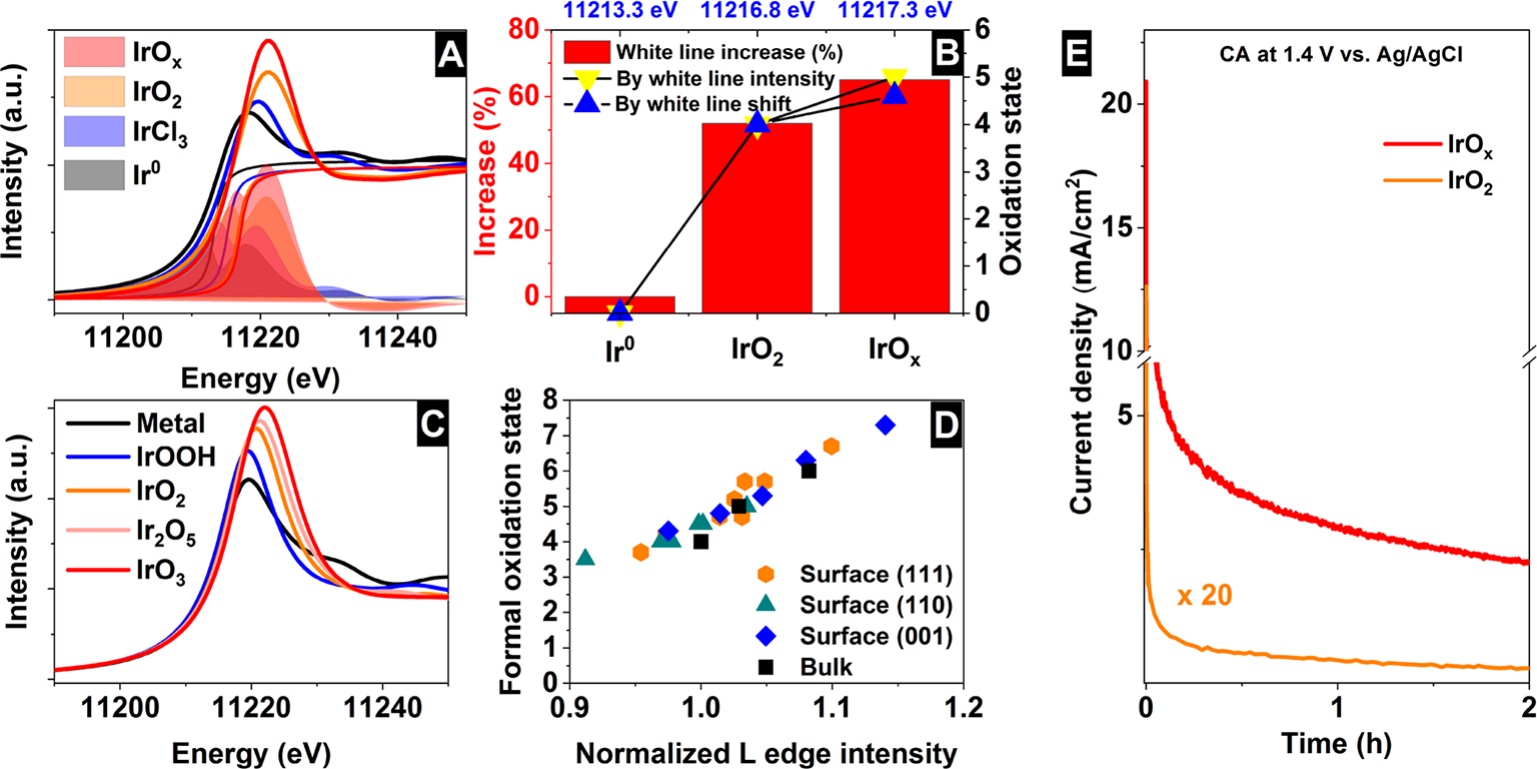
(A) Ir L_3_ spectra of different reference samples including the background subtraction and remaining signal related to the sum-rule of the Ir 5d electron-holes. (B) Increase of integrated area relative to IrCl_3_ and absolute oxidation state deduced from the relative increase of the area or the white line position. Calculated (C) Ir L_3_-edge spectrum of the surface and bulk of an iridium oxides (see Figure S10 for results including a Hubbard *U*) and (D) correlation between computed L_3_-edge maximum intensity and formal oxidation state. (E) Catalytic performance comparison of the IrO_*x*_ and IrO_2_ at 1.4 V vs Ag/AgCl in deaerated 100 mM H_2_SO_4_ with N_2_ continually bubbled in the electrolyte.

Figure 3A shows the Ir L_3_ spectra of the reference samples including the background subtraction and remaining signal, which relates the number of Ir 5d holes to the integral area of the white line.^48^ These measurements clearly show an increase in the Ir L_3_-edge white-line intensity for the different samples following the trend: Ir^0^ < IrCl_3_ < IrO_2_ < IrO_*x*_. Moreover, in Figure 3B the maximum intensity of the white-line is linearly correlated with the integral intensity and, therefore, Ir oxidation state. Thus, for simplicity we employ the maximum white-line intensity as a measure of the Ir oxidation state. Similarly, the Ir L_3_-edges computed using the Bethe–Salpeter equation show the same trend continues through the bulk oxides Ir_2_O_5_ and IrO_3_ (Figure 3C,D); see SI for more details (Figure S4). From the white-line integration of the experimental data, Figure 3B shows the percent increase in the Ir 5d density of electron-holes referred to IrCl_3_ and the equivalent oxidation state. Therefore, an oxidation state of +4 corresponds to an increase of ∼22% of the white-line intensity with respect to +3 (IrCl_3_ sample), and a change of ∼32% for the case of IrO_*x*_ corresponding to a +4.7 average oxidation state. This increase is supported by its comparison with Ir^0^, where an increase of 52% of the white-line intensity is found for +4 and 65% for IrO_*x*_, corresponding to a +4.6 average oxidation state. Performing a similar quantification using the simulated data shows the maximum white-line intensity changes linearly with the Löwdin charge on Ir in the bulk phases as well as across various surface Ir species bound to O, OH, OOH, and OO on IrO_2_ surfaces spanning formal oxidation states from <IV to >VII (see SI for details, Figures S5 and S6; also note these results are incentive for the inclusion of a Hubbard *U*; see Figure S10), suggesting no significant changes in intensity variation should be expected at higher oxidation states for the iridium oxyhydroxides. One can also consider that the change in the oxidation can often be correlated to the shift in the peak position, using this approach an average experimental oxidation state of +4.3 is found for IrO_*x*_, similar to the integration of the white line (Figure 3C). Both approaches yield an average oxidation state for IrO_*x*_ under vacuum >IV suggesting the presence of Ir^V^, in agreement with the Ir^V^ reported to appear in IrO_*x*_ during reaction conditions using a similar analysis, but significantly less than the Ir^VI^ reported^11^ to appear in Ni-leached IrNiO_*x*_. According to Figure 3E it is clear that the formation of such active species has a positive impact in the electrocatalytic performance of the sample containing more Ir 5d holes. However, the increased amorphization of the IrO_*x*_ surfaces had been ascribed as a possible reason for the increased electrocatalytic activity during the OER,^56,57^ which can lead to the formation of Ir^V^ active sites. Thus, the higher white-line intensity of the NPs compared to the thin films is likely due to incomplete oxidation of the bulk in thick films.^58,59^ To see if evidence for such high oxidation state Ir emerges for Ir NPs and thin films under reaction conditions, we return to *in situ* measurements.

**Figure 4:**
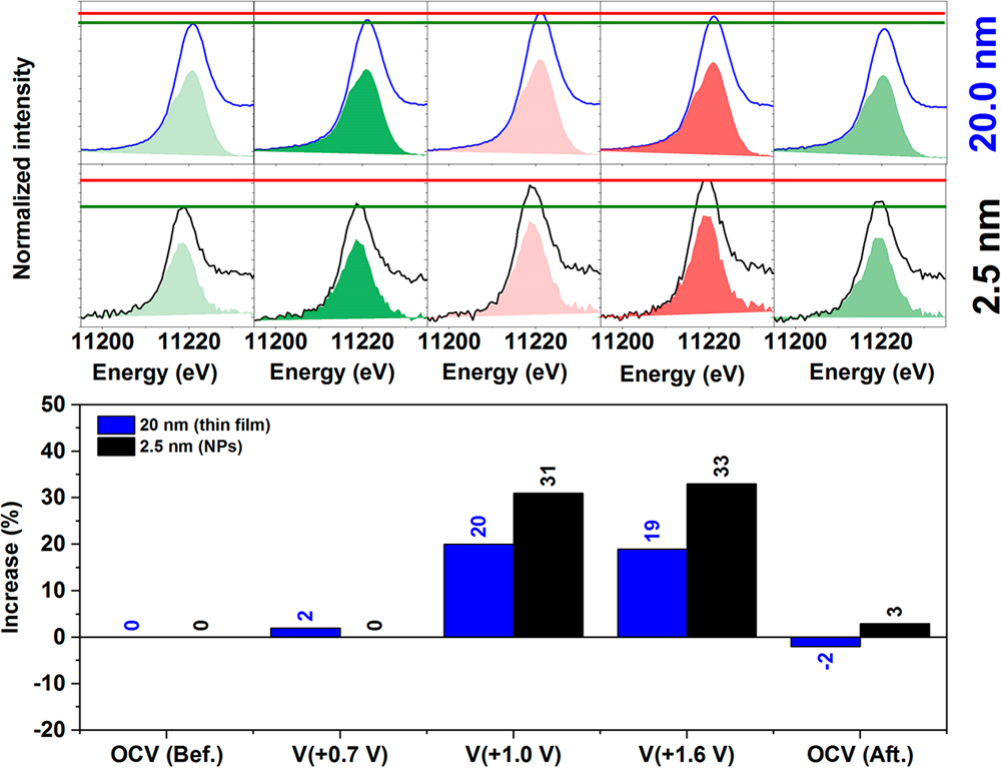
Top: Spectra at different potential for the thin-film IrO_*x*_ electrode (20 nm) and free-standing CVD bilayered graphene (BLG) electrode decorated with IrO_*x*_ 2.5 nm NPs (top). Bottom: Relative increase of the hole density against the electrode potential recorded during the *in situ* characterization.

Figure 4 shows how the measured white-line intensities change with applied bias referred to the OCV spectra, which is used as reference. The spectra were collected under chronoamperometric (CA) control, and ∼1 h was required to record each Ir L_3_-edge spectrum. This fact, together with the constant current observed under the applied potential, verifies that the spectra where collected under steady state conditions, which is important to ensure their fidelity. The surfaces likely remain partially oxidized after the CV activation steps^60^ making an assignment to Ir^III^ more appropriate, as a consequence of the surface irreversible oxidation state, as confirmed by XPS below. At 1.0 V, Ir^IV^ has become the oxidation state, which is further supported by the CVs of the film and NPs showing 1.0 V the transition assigned to Ir^IV^/Ir^V^. At 1.0 V the near-surface region is then expected to be Ir^IV^. The higher white-line intensities confirm oxidation, with the ∼30% increase for the Ir NPs in line with an increase to +4.5, assuming Ir^III^ to start. The higher white-line intensity of the NPs compared to the thin films is likely due to incomplete oxidation of the bulk of the 20 nm thin films.^58,59^ At 1.6 V, though no further increase in white-line intensity is observed for the thin films, the NPs show an additional ∼4% increase above that observed at 1.0 V suggesting the coexistence of Ir^IV^/Ir^V^, or higher oxidation states, on the catalyst surface. The reverse scan back to OCV shows the process reversibility. To gain a better understanding of what these species may be, we turned to *ab initio* calculations.

**Figure 5:**
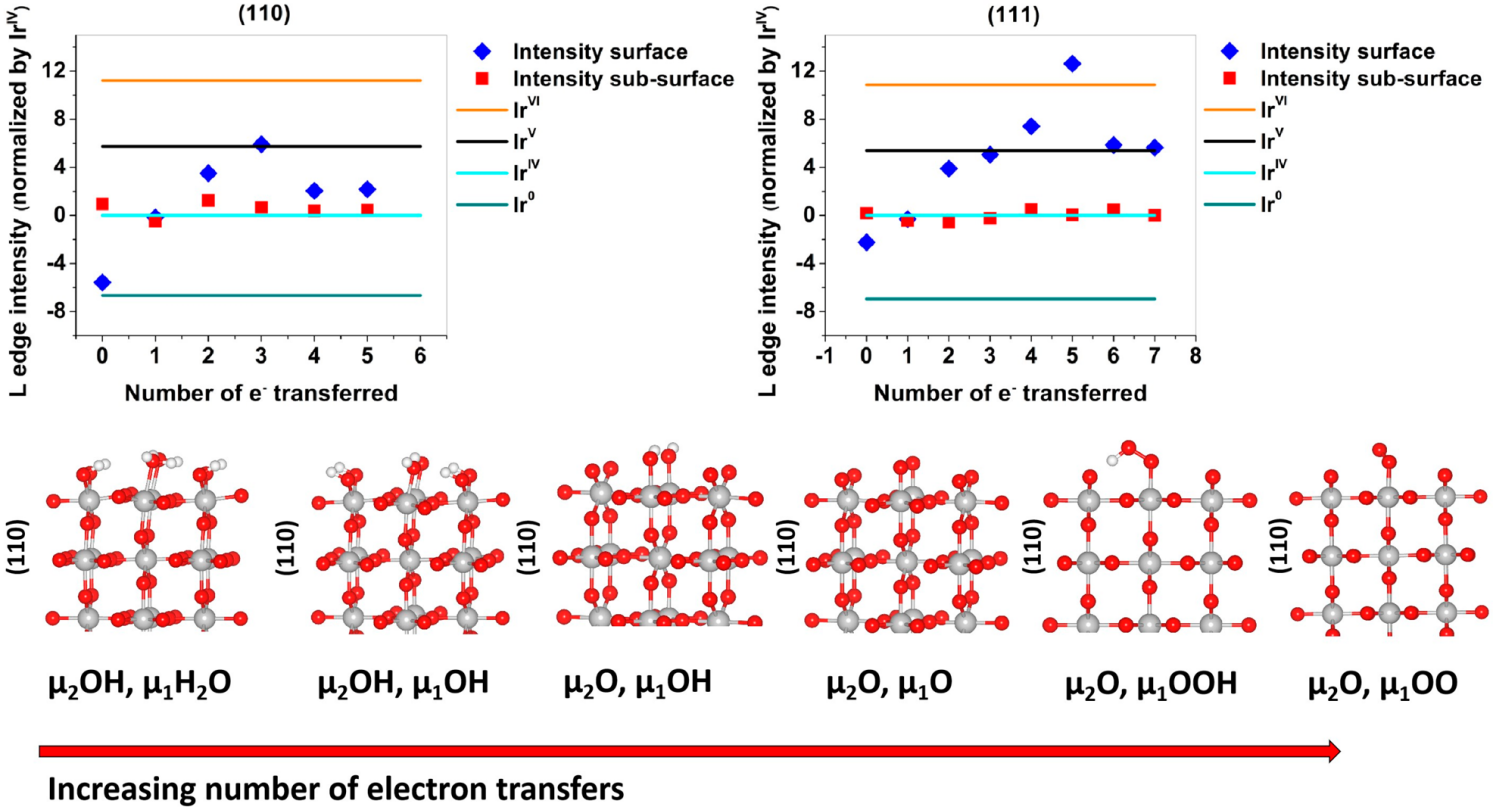
Plots of L_3_ intensity vs electron transfers for (110) and (111) surfaces of IrO_2_. On the bottom the different figures are shown for the (110) surfaces. (111) structures are shown in the SI.

In order to provide a better description of the observed changes in the white-line spectra, these reference and *operando* measurements were compared with the DFT calculated Ir L_3_-edge spectrum (details can be found in the SI). We begin with the lowest energy, in vacuum, IrO_2_ surface, (110), as a model for the DFT calculations.^60^ The surface was first fully hydrogenated as shown in Figure 5. It was then successively oxidized by following a series of proton coupled electron transfers to explore the adsorbates argued to be present under OER conditions;^60^ see Figure 5. The Ir L_3_-edge white-line intensities of these surfaces were computed and plotted against the number of electrons transferred from the hydrogenated surface unit cell to reach the desired surface oxidation state. From Figure 5 it can be seen that only the surface Ir atoms respond to the oxidation, with the first subsurface layer already converged to the L_3_-edge white-line intensity of bulk Ir^IV^. The average surface oxidation state can be seen to increase from a white-line intensity below Ir^IV^ when μ_1_-H_2_O and μ_2_-OH are present—where the subindex indicates the number of iridium atoms bound to an oxygen atom—to a value consistent with bulk Ir^V^ when μ_1_-O and μ_2_-O are present. The former is close to the +3.5 average formal oxidation state for the surface Ir atoms found by simple bound counting.

Removing H^+^ and e^–^ from μ_1_-H_2_O on the (110) surface yields a surface with μ_1_-OH and μ_2_-OH that is predicted to be stable up to 0.7 V.^60^ The average formal surface oxidation state of Ir on this surface is +IV, in-line with the computed L_3_-edge white-line intensity, Figure 5. At 0.7 V the surface termination is predicted to transform into μ_1_-OH and μ_2_-O, with an average Ir surface oxidation state of +4.5, in general agreement with the experimental results on the NPs. Above 1.2 V both a surface μ_1_-O or μ_1_-OO have been predicted to be stable.^39,60^ Of these, the μ_1_-O appears more likely on the NP surface owing to the small increase in white-line intensity observed experimentally between 1.0 to 1.6 V, which is consistent with the increase in iridium formal oxidation state and the computed white-line intensity for transitioning from μ_1_-OH to μ_1_-O. The μ_1_-OO (and μ_1_-OOH) show reduced white-line intensities relative to μ_1_-OH and μ_1_-O, and while we observed no evidence for such a reduction in intensity, it has been previously observed on IrO_*x*_ NPs at high applied potential.^61^

We also considered the possibility that the NPs facet during anodic polarization, as above 1.1 V the (111) surface is thermodynamically favored.^60^ Following the same methodology as above, Figure 5 shows the Ir L_3_-edge white-line intensities as a function of the number of electrons transferred starting from a fully hydroxylated (111) surface; see SI for structures (Figure S5). Here, though more points are included owing to the presence of four μ_2_-OH species in the unit cell, a similar trend emerges as found for the (110) surface, with the Ir L_3_-edge white-line intensities reaching higher values due to the increased formal surface iridium oxidation states on the (111) vs (110) surface. In particular, the Ir bound to μ_1_-O reaches a formal oxidation state exceeding Ir^VI^. Such a species is predicted to appear once the bias reaches 1.1 V; above 1.2 V μ_1_-O may transform into a μ_1_-OO.^60^ While we see no drop in white-line intensity on the NPs supporting the formation of μ_1_-OO, we cannot completely rule out the appearance of Ir^VI^ from XAS alone. Thus, we turned to XPS, as this technique apart from providing complementary information on the occupied orbitals is also more sensitive to the surface states.

**Figure 6:**
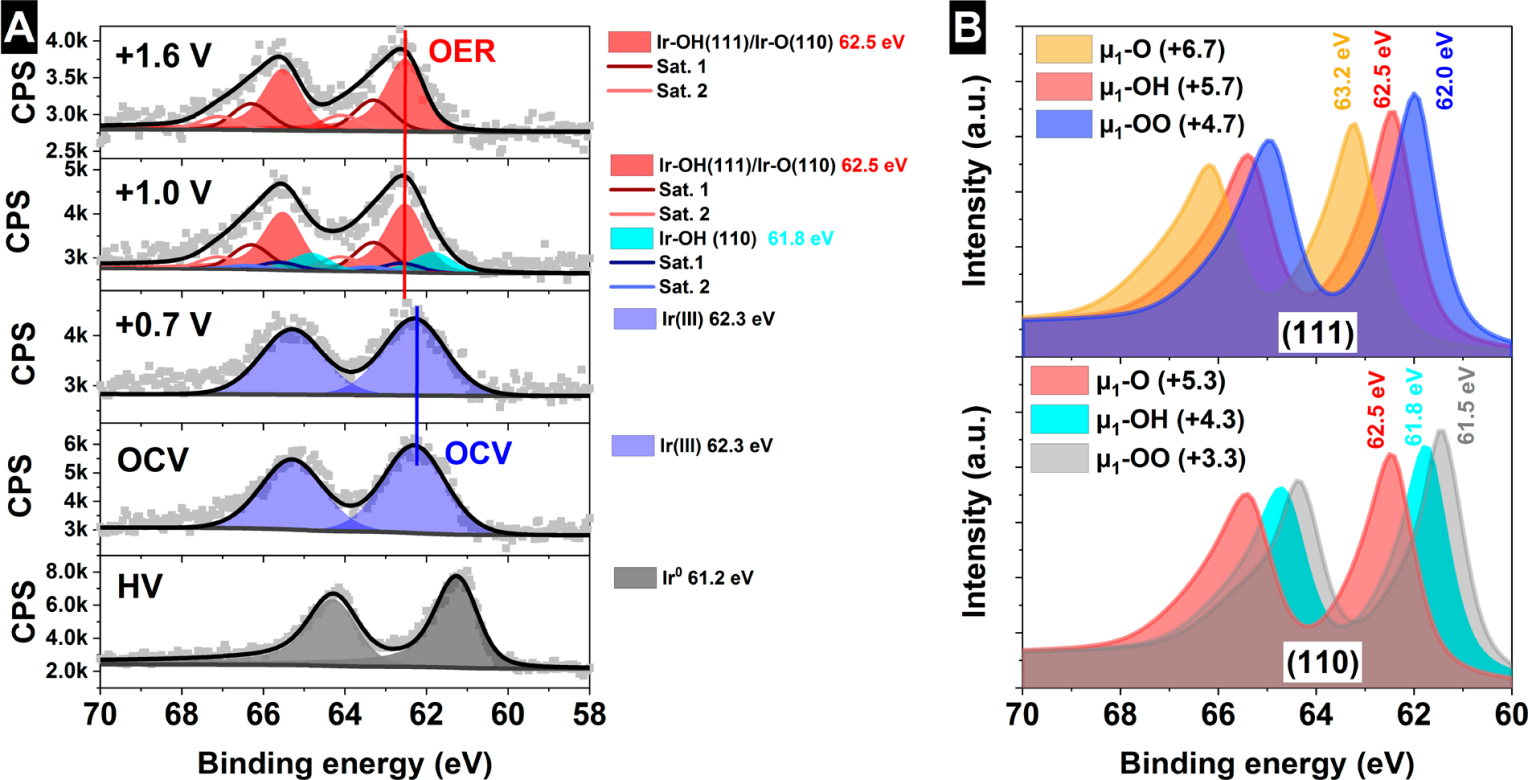
*Operando* measurements in 100 mM H_2_SO_4_ of the free-standing bilayer graphene decorated with IrO_*x*_ NPs at different potentials vs Ag/AgCl (A). Ir 4f (B) simulated Ir 4f spectra of species identified in the Ir L_3_ edge analysis with the formal oxidation state in brackets.

As a surface informative technique, XPS compliments the bulk sensitive XAS and offers the opportunity to gain deeper knowledge about the mechanism mediating the electrochemical oxidation of water on iridium oxide. The electronic structure of the IrO_*x*_ NPs on graphene was investigated at the Ir 4f core level using the facilities and *in situ* electrochemical flow cells described in the SI.^44^ The Ir 4f spectra depending on the applied potential are shown in Figure 6A, as well as its comparison with the reference samples (bottom spectra) and the computed line shapes for the relevant species inferred through the XAS analysis (see Figures S7 and S8). Computed XPS binding energies are summarized in Table S1.

At, and below, 0.7 V the NPs are composed of predominantly bulk Ir^III^, with an Ir 4f binding energy of 62.3 eV.^62^ Once the electrode is polarized to 1.0 V, we know from XAS that the NPs are oxidized to Ir^IV^, and possibly higher. Note that the lack of a linear shift in the measured Ir 4f with applied potential indicates good electrical contact between the graphene and Ir NPs. In addition, the change in the spectrum shape is in good agreement with a close interaction with the electrolyte and correct electrochemical performance, i.e. no gas-bubble accumulation on the electrode surface hindering the electrical contact. From the simulations we find that Ir atoms on the surface of IrO_2_ show normal Ir 4f binding energy shifts up to formal Ir oxidation states of, at least, 7.3 (see Table S1), with the average formally Ir^V^ and Ir^VI^ species appearing near ∼62.2 eV and ∼62.7 eV, respectively. Thus, the Ir 4f shift is a good measure of the iridium oxidation state for Ir atoms in a conductive matrix. However, this conductive matrix also influences the Ir 4f line shape, making accurate speciation challenging. The computed Ir 4f spectra all show complex line shapes due to conduction band screening (see Figure 6B for computed XPS), similar to that observed in rutile-type IrO_2_,^10,63^ where shakeup satellites are required to fit the spectral envelope. The line shape varies systematically with the formal oxidation state of Ir, with the primary satellite shifting closer to the main line with increasing iridium oxidation state; see Figure 6B. Noting this behavior allows a suitable fit model to be developed.

Using this fit model we see that, at an applied bias of 1.0 V, peaks appear at 61.8 and 62.5 eV, both of which show strong conduction band screening. The first of these peaks is consistent with the Ir^4.3^ bound to μ_1_-OH on the (110) surface. The species at 62.5 eV is consistent with the Ir^5.3^ bound to μ_1_-O on the (110) surface or the Ir^5.7^ bound to μ_1_-OH (or μ_1_-OOH) on the (111) surface. Thus, at 1.0 V the near-surface region appears to be dominated by Ir^IV^/Ir^V^ and μ_1_-OH/μ_1_-O. These findings are in general agreement with predictions based on *ab initio* atomistic thermodynamics.^60^ Details of the parameters used for fitting the spectra can be found in the SI (Table S2), where a Shirley^64^ background is used. Note the fact that only a minimal contribution of remaining Ir^IV^ species were detected in the Ir 4f peaks under OER, indicating that this signal is produced by atoms buried in the bulk of the catalyst (i.e., not reacting with the water molecules in the electrolyte). Meanwhile, the Ir^V^ signal comes mostly from Ir atoms located in the surface and near-surface of the nanoparticles. Thus, the combination of nanostructured electrodes with surface informative photoelectron spectroscopy is an effective tandem for increasing the information on the electrified interface during the electrocatalytic processes, which can be applied to other reactions of interest.

Under OER (−1.6 V vs Ag/AgCl) conditions the species at 62.5 eV becomes the dominant species and no higher binding energy component is found. This suggests that a large fraction of the high oxidation state species is present, being not specific to a unique potential, as the bias increased from 1.0 to 1.6 V vs Ag/AgCl, as shown in Figure 6A, in agreement with the XAS-TFY *operando* measurements. This rules out this species as a reactive intermediate, because it would already react and produce oxygen at −1.0 V vs Ag/AgCl. With XPS, however, we can rule out the possibility of Ir oxidation states of Ir^VI^ and above since these would appear at higher binding energies than those observed. Thus, taking XPS and XAS together we tentatively assign the species appearing at 62.5 eV to a formally Ir^V^ species bound to μ_1_-O on the (110) surface. While small amounts of the Ir at this high binding energy has also been observed in both *ex situ*(65) and *in situ*(66) studies, the surface model system employed in this work allows us to show the formally Ir^V^ species is the dominant surface species under the OER (−1.6 V vs Ag/AgCl), in good agreement with previous results,^66^ a key aspect in materials with a high surface-to-bulk ratio.^67^ Unlike the previous XPS experiments performed in gas-phase conditions,^66^ the results reported herein were conducted in the presence of bulk aqueous electrolyte. The enhanced changes in the spectra observed in this work may then be a consequence of better interface hydration. The performance of the water vapor *in situ* cell and the quality of the spectra collected were significantly improved in our group in recent years.^40,68−70^ However, these experiments were not performed in the presence of a bulk aqueous electrolyte, which can potentially yield different results from those found in a typical three-electrode benchtop experiment. In addition, the experimental conditions reported here are collected under the same environmental conditions than in the case of the *operando* XAS experiments making possible a direct comparison of the results obtained with both approaches. It is not doable between the different studies present in the literature because they combine different catalysts with different EC-cells designs, electrolytes, and experimental conditions making a direct comparison between them undesirable. When combined with *ab intio* simulation, this allows a more definitive assignment to be made. Moreover, from the simulations we see that, at such high formal Ir oxidation states, the μ_1_-O species bound to Ir^V^ may also be referred to as O^I-^ owing to the spin density on oxygen.^71^ This electron deficiency on μ_1_-O makes the oxygen susceptible to nucleophilic attack,^11,39,63,72^ suggesting the high activity of the Ir NPs is linked to electron-hole enrichment on μ_1_-O.

**Figure 7:**
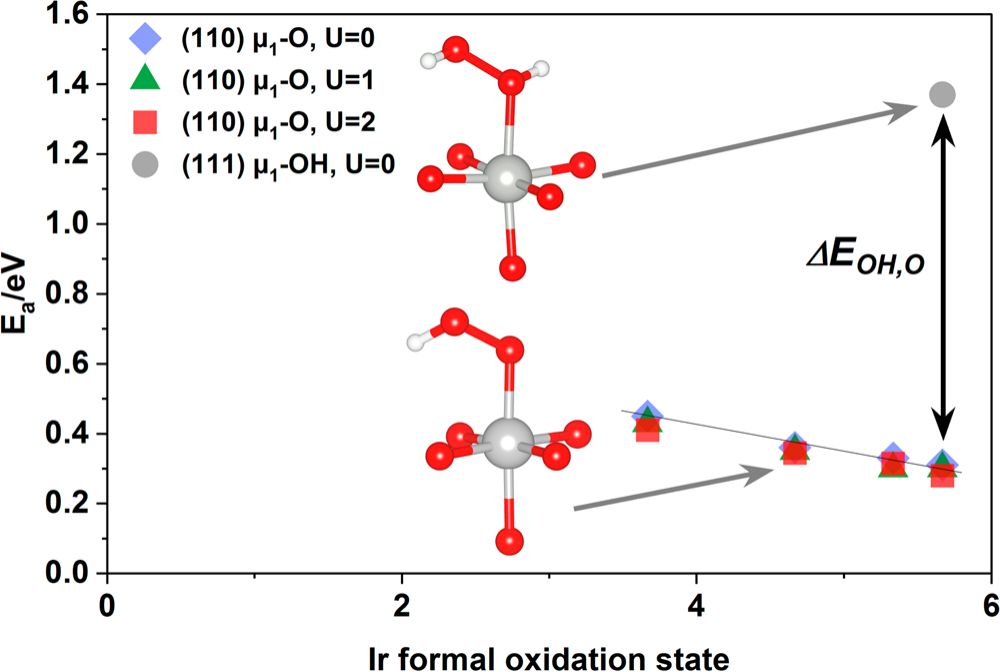
Heat of reaction computed for O−O bond formation between H_2_O on the (111) surface and μ_1_-O(H) on the (110) surface. The heat of reaction represents the minimum activation energy for the reaction. The Ir(V)–OH on the (111) surface reacts to produce bound HOOH and shows a high activation energy despite the high formal Ir oxidation state. On the (110) surface the Ir(V)–O reacts with H_2_O to form μ_1_-OOH and shows an activation energy more than 1 eV lower than that associated with Ir(V)–OH, the difference labeled Δ*E*_OH,O_. Varying the oxidation state of Ir bound to μ_1_-O results in a linear change in the computed activation energy. See also Figure S9.

To verify the reactivity of the μ_1_-O(H) and the role of iridium oxidation state on electrocatalytic performance, we computed the activation energy for O–O coupling on the (110) surface while varying Ir oxidation state within the range observed experimentally. This step was chosen, as it is believed to be rate limiting^72^ and can be approximated as a chemical step wherein an O–O bond is formed between μ_1_-O and H_2_O.^73^ (Note that while the exact values of the these energies may differ with solvent, their trend is expected to remain qualitatively correct.^73^) Figure 7 shows the computed activation energies for O–O bond formation through this process on the (110) surface plotted as a function of formal Ir oxidation state, along with the corresponding heat of reaction between H_2_O and Ir(V)/μ_1_-OH on the (111) surface, which is the lower bound on the activation energy; see Figure S9 for structures. Inspection of Figure 7 reveals O–O bond formation between H_2_O and Ir(V)/μ_1_–OH on the (111) surface is slow, with a thermodynamic barrier in excess of 1.4 eV associated with the formation of the adsorbed HO–OH shown in Figure 7. This picture contrasts the high activity of Ir(V)/μ_1_-O on the (110) surface. In this case, the activation energy to O–O coupling is only 0.33 eV because the hole character on μ_1_-O facilitates O–O bond formation. Thus, Ir oxidation state alone does not control the activation energy for O–O coupling, and electron-deficient oxygen appears to be needed. When electron-deficient μ_1_-O is present, however, varying the oxidation state of the iridium bound to the μ_1_-O involved in O–O coupling shows the Ir oxidation state does play a prominent role. In Figure 7 this was done by introducing a surface Ir vacancy, subsurface O vacancy, or substituting subsurface O with H; see Figure S9. The result is that the computed activation energy drops almost linearly with increasing formal Ir oxidation state; see Figure 7. Introducing a Hubbard *U* correction does not alter this observation. Thus, it appears the μ_1_-O that can appear when the Ir oxidation state reaches V+ facilitates O–O bond formation and its activity is sensitive to the Ir oxidation state.

With this picture of the catalytic performance, it is useful to return to the measured spectra. When the XPS and XAS measurements are compared an important aspect of the active state of the material can be seen. The relative thickness of the electron-hole enrichment beyond Ir^IV^ is confined to the near-surface region, while Ir^IV^ may extend through the bulk. XPS shows the surface and near-surface of the NPs are dominated by the formation of active Ir^V+^ (or O^I–^) species that are active in the electrocatalytic oxidation of water. These active species contribute ca. ∼4% to the overall Ir L_3_ white-line intensity observed for the NPs when increasing the bias from 1.0 to 1.6 V vs Ag/AgCl, while the Ir^IV^ bound to μ_3_-O species located in the bulk dominate the ∼30% increase in white-line intensity seen when increasing the bias from 0.6 to 1.0 V vs Ag/AgCl. By contrast, for the 20 nm films, surface oxidation past Ir^IV^ cannot be discerned from the bulk oxidation to Ir^IV^ owing to the overwhelming bulk signal.

## Conclusions

In summary, the combination of *ab initio* calculations, XA and PE spectroscopies, and nanofabrication of thin-film IrO_*x*_ and free-standing graphene decorated IrO_*x*_ NP electrodes provided relevant information related to the active sites of iridium-based electrocatalysts in the kinetically sluggish OER. It was found that the electrocatalytic activity of IrO_*x*_ is ascribed to the formation of formally Ir^V^ species bound to μ_1_-O, where, due to the electron deficiency of these Ir sites, the μ_1_-O on the surface of the electrocatalyst that is susceptible to nucleophilic attack by water. Our results show that the potential-dependent oxidation state changes in the IrO_*x*_ extend through the bulk for oxidation states below Ir^V^ but are constrained to the near-surface region for higher oxidation states, suggesting bulk oxidation is limited. Thus, the higher surface-to-bulk ratio of nanostructured materials enhances iridium usage and the participation of a significant larger amount of surface and near-surface active μ_1_-O. In addition we provide evidence using bulk electrolyte for these changes using surface informative XPS, which is in good agreement with the bulk sensitive XAS-TFY measurements yielding a direct link between surface and bulk effects in electrocatalytic OER. The different contribution of bulk and surface was discriminated, where the bulk maintains Ir^IV^ character, while the surface undergoes oxidation to Ir^V^.

## Supporting Information Available

Experimental details including sample and electrolyte preparation, reference samples, instrumental details, *operando* electrochemical cells description, beamlines and DFT calculation details. Pictures of the *operando* electrochemical cells. Supplementary figures including SEM images of the free-standing graphene electrode, balls and sticks structure-models used for the DFT calculations, DFT calculations of the Ir 4f main peak and satellite position depending on the oxidation state, photoelectron spectra of the transmission properties of the free-standing graphene. Tables including computed and XPS fitting parameters (PDF)
