# Supplementary material for: Surface Electron-Hole Rich Species Active in the Electrocatalytic Water Oxidation
Source: J Am Chem Soc. 2021 Aug 6;143(32):12524–34. doi: 10.1021/jacs.1c01655 (PMC8397309; doi:10.1021/jacs.1c01655)
Supplement: Supplementary file 1 — ja1c01655_si_001.pdf [file ja1c01655_si_001.pdf]

Supplementary Information

Surface electron-hole rich species active in the electrocatalytic water oxidation

Juan-Jesús Velasco-Vélez[1,2,*], Emilia Carbonio[2,3], Cheng-Hao Chuang[4], Cheng-Jhih Hsu[4], Jyh-Fu Lee[5], Rosa Arrigo[6], Michael Hävecker[1,2], Ruizhi Wang[7], Milivoj Plodinec[2,8], Feng Ryan Wang[9], Alba Centeno[10], Amaia Zurutuza[10], Lorenz J. Falling[2], Rik Valentijn Mom[2], Stephan Hofmann[7], Robert Schlögl[1,2], Axel Knop-Gericke[1,2], Travis E. Jones[2*]

***Corresponding author:** velasco@fhi-berlin.mpg.de, trjones@fhi-berlin.mpg.de

[1]Department of Heterogeneous Reactions, Max Planck Institute for Chemical Energy Conversion, Mülheim an der Ruhr 45470, Germany

[2]Department of Inorganic Chemistry, Fritz-Haber-Institut der Max-Planck-Gesellschaft, Berlin 14195, Germany

[3]Helmholtz-Center Berlin for Materials and Energy, BESSY II, Berlin 12489, Germany

[4]Department of Physics, Tamkang University, New Taipei City 25137, Taiwan

[5]National Synchrotron Radiation Research Center, Hsinchu 30076, Taiwan

[6]School of Sciences, University of Salford, Environment and Life, Cockcroft building, M5 4WT, Manchester, U.K.

[7]Department of Engineering, University of Cambridge, Cambridge CB3 0FA, United Kingdom

[8]Rudjer Boskovic Institute, Bijenicka 54, HR-10000 Zagreb, Croatia

[9]Dept. Of Chemical Engineering, University College London, Torrington Placa, WC1E7JE, UK

[10]Graphenea, San Sebastian 20018, Spain

Electrode preparation. Graphene was grown by chemical vapor deposition (CVD) on a 20 μm thick Cu foil (Alfa Aesar 99.8%) as catalysts and CH_4 (diluted in Ar and H_2) at 1000 °C using an Aixtron BM Pro (4 inch) reactor yielding a continuous polycrystalline film with grain size in the range of ~20 μm, which was confirmed by scanning electron microscopy. The graphene layer was fixed to 500 nm of Poly(methyl methacrylate) PMMA (4 wt.% in anisole, 950k molecular weight) deposited by spin coating. After that, the copper support was eliminated by floating on a 50 mM aqueous solution of $(NH_4)_2S_2O_8$. The graphene/PMMA layer was floated in deionized water (DI-water) and transferred onto another graphene/copper foil and dried at 50 °C for 5 minutes. The resulting sample was floated again in the $(NH_4)_2S_2O_8$ solution to remove the copper foil before rinsing in DI-water. The PMMA/BLG layer was transferred to a Norcada® Si_3N_4 TEM grid with 500 nm diameter holes or onto a SiN_x membrane 100 nm thick for the TFY measurements[1,2]. Finally, the PMMA was eliminated with acetone and the adherence between the BLG and the substrate is due to Van der Waals interaction ensuring stability for the electrodes. It produces a continuous film that can be used as an electrode in aqueous environments as electrocatalytic applications among others[3,4].

Fig. S1 A and B show the SEM measurements of the free standing graphene on the Si_3N_4 grid. The influence of the defects in the graphene layer (i.e. holes, tears etc) is suppressed by the

addition of a second graphene layer. Raman spectroscopy was used to check the lattice vibrations of the crystallized BLG which is sensitive in order to determine the graphitic character of this sample. Fig. S1 C shows the Raman spectra of different reference samples such as HOPG, single layer graphene (G), graphene oxide (GO), reduced graphene oxide (RGO), and the transferred BLG used in this study. The origin of the graphitic Raman spectrum is well established and is accepted that it presents three first order bands between 1000 cm^{-1} and 3000 cm^{-1}: D band at ~1350 cm^{-1}, G band at ~1580 cm^{-1} with a shoulder at ~1620 cm^{-1}.[5,6] The D band is associated with defects that perturb the breathing modes of carbon rings. The G band is due to the in-plane phonons at the Brillouin zone centre. The 2D band is due to excitation of two phonons with opposite momentum in the highest optical branch near the K point and is sensitive to the number of graphene layers[7]. The Raman spectrum of HOPG does not show a D band, which attributed to the small number of points defects, and attenuated 2D due to the excitation of two phonons with opposite momentum in the underlying layers. On the other hand, the graphene reference spectrum does not show a D band and the ratio of the 2D and G peak intensities, i.e., I_{2D}/I_G is approximately ~2 indicating a good graphitization and likely the absence of more underlying layers[21]. Graphene oxide (GO) shows a I_{2D}/I_G ratio decreases and the D peak intensity increases, indicating an increase in the defect concentration. This trend in the peak intensity is not due to the presence of oxygen, as the similar behavior of the reduced graphene oxide (RGO) spectrum proves, indicating that the origin of this behavior is the existence of defects in the graphene lattice. The absence of the D band confirms the BLG is of high-quality with a low density of defects in the graphene lattice. In addition, the reduction in the I_{2D}/I_G ratio to approximately ~1.7 suggests the presence of an underlying graphene layer due to the excitation of phonons with opposite momentum, which reduces the intensity of the 2D.

The electrodes were deposited by sputtering using Cressington 208HR sputter coated machine loaded with an Ir target (99.99% from Elektronen-Optik-Service GmbH, Germany) in a 0.1 mbar Ar atmosphere at a current of 40 mA. The sputter time determined the thickness of the electrode: the nanoparticles were deposited for 5 s yielding the formation of a homogeneous well distributed Ir NPs in the whole surface with NPs size ranging from 2 to 5 nm in the form of metallic iridium (Ir^0). The 20 nm thin-film electrodes were fabricated by sputtering onto a SiN_x membrane (100 nm thick and 5 mm x 5 mm, with a Si frame of 1 cm x 1 cm and 200 µm thick sourced from Norcada) in a 0.1 mbar Ar atmosphere at a current of 40 mA for 160 s (see Fig. 1A). There are stoichiometric iridium oxide NPs that remain unaltered under the electron beam, being more stable than the non-stoichiometric iridium oxide (Ir_xO_y) nanoparticles. These particles are in the form of IrO_2 rutile with orientation (210) and 0.20 nm lattice constant and IrO_2 (201) and lattice plane distance of 0.26 nm both with tetragonal crystal structure, as shown in Fig. 1B. At higher magnification it was found that some of the iridium NPs are reduced to Ir^0 under vacuum conditions and in presence of the electron beam. It is a well know problem and is related to the existence of a non-stoichiometric iridium oxide (Ir_xO_y) giving Ir^0 (111) cubic structure with a lattice plane distance of 0.23 nm as a product of the reduction by the beam. The thickness of the electrode was calibrated for the different sputter time, pressure and current

conditions using cross section images acquired with an electron microscope. We found this procedure is more accurate than the readings provided by the micro-quartz balance in the sputtering chamber.

The electrodes were oxidized/activated for their use in OER by potential cycling by continuous cyclic-voltammogram (CV) between 0 V and 1.2 V vs. Ag/AgCl (DRIREF-2SH, World Precision Instruments, US) and Pt counter electrode for 10 minutes in 0.1 M of H_2SO_4 (EMSURE 97%, Merck, Germany) in Mili-Q water (18.2 MΩ) at a rate of 20 mV/s at room temperature (RT), 25°C. The continuous cycling between open circuit voltage (OCV) and OER in a H_2SO_4 electrolyte at RT results in the electrode activation.

***In situ* electrochemical cell.** The EC-cell used for hard X-ray measurements is shown in Fig. S2A, where the flow of liquid was assured with a peristaltic micro pump. This cell is based on a 100 nm thick SiN_x membrane, transparent to the incoming X-ray photons, possible photon-in/photon-out (PIPO) techniques possible in the hard X-ray regimen, thereby enabling the study of electrochemical reactions with aqueous electrolyte. The SiN_x window is 100 nm thick, with an area of 5 mm x 5mm and silicon frame of 1 cm x 1 cm and 200 µm thick[9].

For the *in situ* XPS characterization, the liquid flow cell[10,11] was operated inside the main chamber of the AP-XPS endstation of the ISISS of the synchrotron facility BESSY II (Berlin, Germany) under a main chamber of $\sim10^{-7}$ mbar background pressure while aqueous electrolyte (0.1 M H_2SO_4) is flowed (1 ml/min) on the back side of the free standing graphene membrane described above, which is used as the working electrode. The continuous flow of liquid was assured by a syringe pump, 260D Teledyne Isco (Lincoln, USA). The main body of the electrochemical cell is made of polyether ether ketone (PEEK), which is an exceptional electrical insulator and chemically inert. The sealing was assured with several Kalrez O-rings, which have good chemical stability. The cell contains two additional electrodes, the counter- (platinum) and reference-electrode (Ag/AgCl DRIREF-2SH, World Precision Instruments, USA). A cross-section of the cell is depicted in Fig. S2B. The Ir 4f spectra were collected with a kinetic energy of the photoelectrons of 600 eV at different potentials. As far as the minimum kinetics energy needed for the photoelectron to penetrate the graphene electrode and reach the detector is 500 eV (see Fig. S3). Thus the collected spectra at 600 eV excitation energy are surface informative (equivalently at around ~100 eV excitation energy in membrane free systems). At open circuit potential (OCP) the Ir 4f peak consists of two main peaks ascribed to the spin orbit split Ir 4f7/2 and Ir 4f5/2 states.

Beamlines. *In situ* synchrotron radiation based experiments were performed at the ISISS beamline of BESSY II in Berlin (Germany). In this facility, the photons are sourced from a bending magnet (D41) and a plane grating monochromator (PGM) yielding an energy range from 80 eV to 2000 eV (soft X-ray range), a flux of $6x10^{10}$ photons/s with 0.1 A ring current using a 111 µm slit and a 80 µm x 200 µm beamspot size. The *operando* measurements were

accomplished in the ambient pressure X-ray photoelectron spectrometer (AP-XPS) end-station of the ISISS beamline, which is equiped with a SPECS PHOIBOS 150 NAP hemispherical analyzer.

Hard X-ray absorption measurements at the Ir L_3-edge were performed at the beamline BL17C1 of the National Synchrotron Radiation Research Center (NSRRC) in Hsinchu (Taiwan). The photons are sourced from a 25 poles wiggler (W20) with 20 cm period length and a focus spot size of 2 mm x 6 mm. The excitation energy ranges from 4.8 keV up to 14.2 keV. The signal was collected in transmission mode for the powder samples and in total fluorescence yield (TFY) mode for the foil and *in situ* EC-cell experiments, using an ionization chamber detector.

Electrolyte preparation. The electrolyte was prepared by diluting 9.8 g of H_2SO_4 (purity 98%, Alfa Aesar, Massachusetts, USA) in 1 L of Milli-Q water (18.2 MΩ) at room temperature (RT), 25 °C. The electrolyte was continuously saturated by bubbling pure N_2 gas, which minimizes the presence of other dissolved gases in the electrolyte. The electrolyte is acidic with pH~1.

Potentiostat. Potentiometric control was assured with a Biologic SP-300 (Seyssinet-Pariset, France) allowing different potentiometric and amperometric controls. The experiments were performed in the presence of aqueous electrolyte where the measurements were acquired using the electrochemically activated IrO_x electrode in presence of 100 mM H_2SO_4 aqueous electrolyte (pH~1) saturated in N_2 (gas) at the same time that the electrolyte was continuously refreshed by a peristaltic pump (Pt counter electrode and Ag/AgCl reference), which minimizes the possibility to form trapped gas bubbles that can potentially influence the electrochemical cell performance.

Reference samples. Commercially available iridium powders were purchased from Sigma-Aldrich (99.9% trace metals basis) and AlfaAesar (Premion, 99.99% trace metals basis). In addition, Ir foil was purchased from Mateck (99.99% metals basis). The AlfaAesar powder was used as received and the SigmaAldrich powder was washed in Milli-Q water and calcined at 800 °C in O_2 for 50 hours. The AlfaAesar powder is X-ray amorphous (IrO_x) wile the calcined sample of SigmaAldrich exhibits the rutile-type structure (IrO_2). More details of the sample preparation/characterization can be found elsewhere[10]. The Ir foil was subjected to several cycles of sputtering in Ar^+ atmosphere and annealing in H_2 atmosphere and shows a purely metallic phase.

Calculations. Density functional theory calculations were performed at the PBE+U level with U=0 using the Quantum ESPRESSO package[12] using pseudopotentials from the PSLibrary[13] with a kinetic energy (charge density) cutoff of 60 Ry (600 Ry). While PBE has been shown to accurately recover the electronic structure of IrO_2, some electron/hole localization beyond this U=0 level may be expected[14]. In fact, using a Hubbard U < 2 eV is known to maintain good agreement between the computed density of states and the measured valence band of iridium

oxide[14] and can also influence surface adsorption energies on iridates[15]. Thus, we also explored the effect of applying a Hubbard U of 1-2 eV to the Ir d orbitals using the simplified rotational-invariant scheme implemented in Quantum ESPRESSO[16]. For bulk rutile-type IrO_2 calculations a (12 × 12 × 12) ***k***-point mesh was used with Marzari-Vanderbilt cold smearing using a 0.01 Ry smearing parameter[17]. The structure for bulk Ir_2O_5 was found using USPEX[18] with Quantum ESPRESSO. A starting population of 28 individuals was employed with a fixed composition of Ir_2O_5. The search was allowed to run until the lowest energy structure remained unchanged for 5 generations. The resulting low energy *C*2/*m* structure from this search was further optimized with Quantum ESPRESSO using a (6 × 6 × 6) ***k***-point mesh and is shown in Fig. S4A. The structure for bulk IrO_3 was taken from the Materials Project (ID:mp-1097041).[19] The orthorhombic *Cmcm* structure was further optimized Quantum ESPRESSO using a (6 × 6 × 6) ***k***-point mesh and is shown in Fig. S4B. Rutile-type (110) surfaces were modeled with symmetric 5-layer slabs separated by 20 Å of vacuum using a (6 × 12 × 1) ***k***-point mesh and 0.01 Ry cold smearing. Rutile-type (111) surfaces were modeled with 11-layer slabs separated by 20 Å of vacuum using a (4 × 4 × 1) ***k***-point mesh and 0.01 Ry cold smearing. Surface calculations included spin-polarization. A 7-layer rutile-type IrO_2 (001) surface was also included to access higher Ir surface oxidation states and was computed using a (6 × 6 × 1) ***k***-point mesh and 0.01 Ry cold smearing with 20 Å of vacuum separating periodic images. Transition states were found using the climbing image nudged elastic band method. The images were relaxed until the force dropped below 0.05 eV/Å and the change in total energy dropped below 0.02 eV between iterations. These simulations were performed using (2 × 1) and (2 × 2) supercells for the (110) surfaces, for the (111) surface, the corresponding heat of reaction was computed using a (2 × 2) supercell.

XAS spectra were computed with a resolvent-based Bethe-Salpeter Equation (BSE) approach[20] using the wavefunctions from Quantum ESPRESSO with the core-level BSE solver in the OCEAN package[21]. For these calculations normconserving pseudopotentials were used with a kinetic energy cutoff of 120 Ry. Empty bands were included to up to 200 eV above the Fermi energy (E_F). Other parameters matched the total energy calculations. All XAS spectra were broadened with a 5.2 eV wide Lorentzian to capture the lifetime broadening at the Ir L_3 edge[22]. Spectra were aligned using ΔSCF calculations[23]. The excited state of each absorbing atom was computed separately using a 2p core-hole on the absorbing atom, and the excited electron was included in the simulation. For bulk IrO_2 ΔSCF calculations were performed using a (4 × 4 × 4) supercell, while for the (110) and (111) surfaces (3 × 6) and (3 × 3) supercells were used, respectively. The spectra were aligned to experiment using bulk rutile-type IrO_2. The effect of a Hubbard U was tested on bulk IrO_2, the (111) surface with μ_1-OH and the (110) surface with μ_1-O. No experimentally observable changes were seen for U≤2 eV, see Figure SIU.

XPS spectra were computed using a Hopfield perturbation model[24]. Following reference[25], the initial density of states (DOS) of the photoexcited atom were taken from the project Ir 5d DOS of the ground state, and the final density of states were taken from the project

Ir 5d DOS of the atom with an Ir 4f corehole using the same supercells as the ΔSCF calculations used for XAS alignment. Constant Gaussian broadening (0.3 eV) was included along with energy dependent Lorentzian broadening. Lorentzian broadening used an empirical Seah-Dench like model[26], where the energy dependent line width above E_F is given by: $\Gamma(E) = \Gamma_0 + \Gamma_{max}[1/2+1/\pi \arctan(e-1/e^2)]$ with $e = (E - E_F)/(E_c - E_F)$. The parameters Γ_{max}, and E_c were taken as 22 and 8 eV, respectively, while Γ_0 was taken as the tabulated natural line width, 0.3 eV.[27] The N_6 and N_7 edges were broadened separately and the spin-orbit splitting was taken from the free atom value. The Ir 4f shifts were computed by way of ΔSCF calculations using the total energy differences from the ground state and excited calculations. The spectra were aligned to experiment using bulk rutile-type IrO_2.

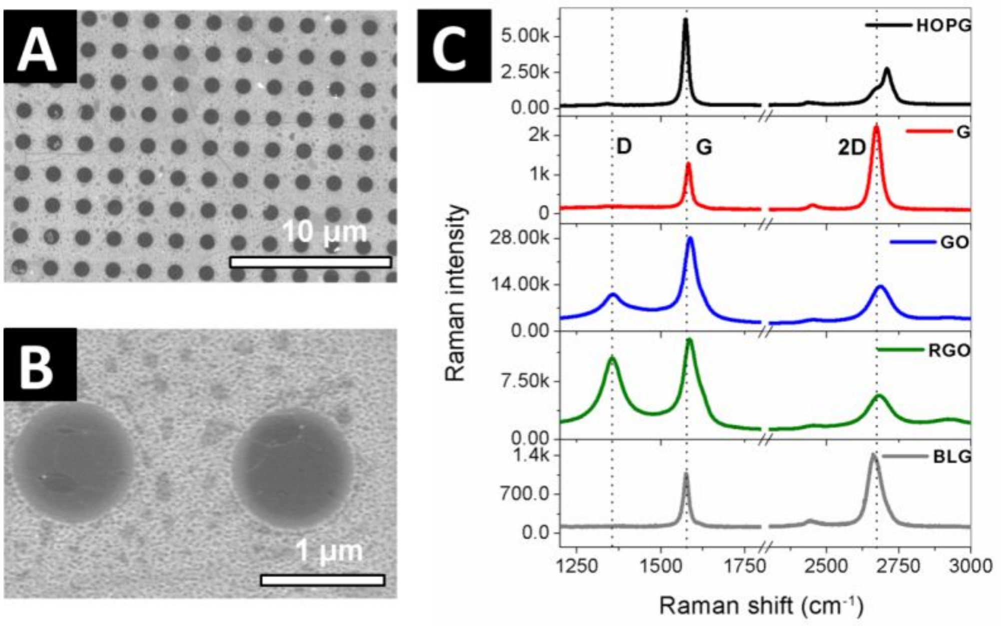

Fig. S1

SEM image of the BLG coating the Si_3N_4 grid with holes of 1 µm diameter: **(A)** 10 µm and **(B)** 1 µm scales. **(C)** Raman spectra of different graphitic materials.

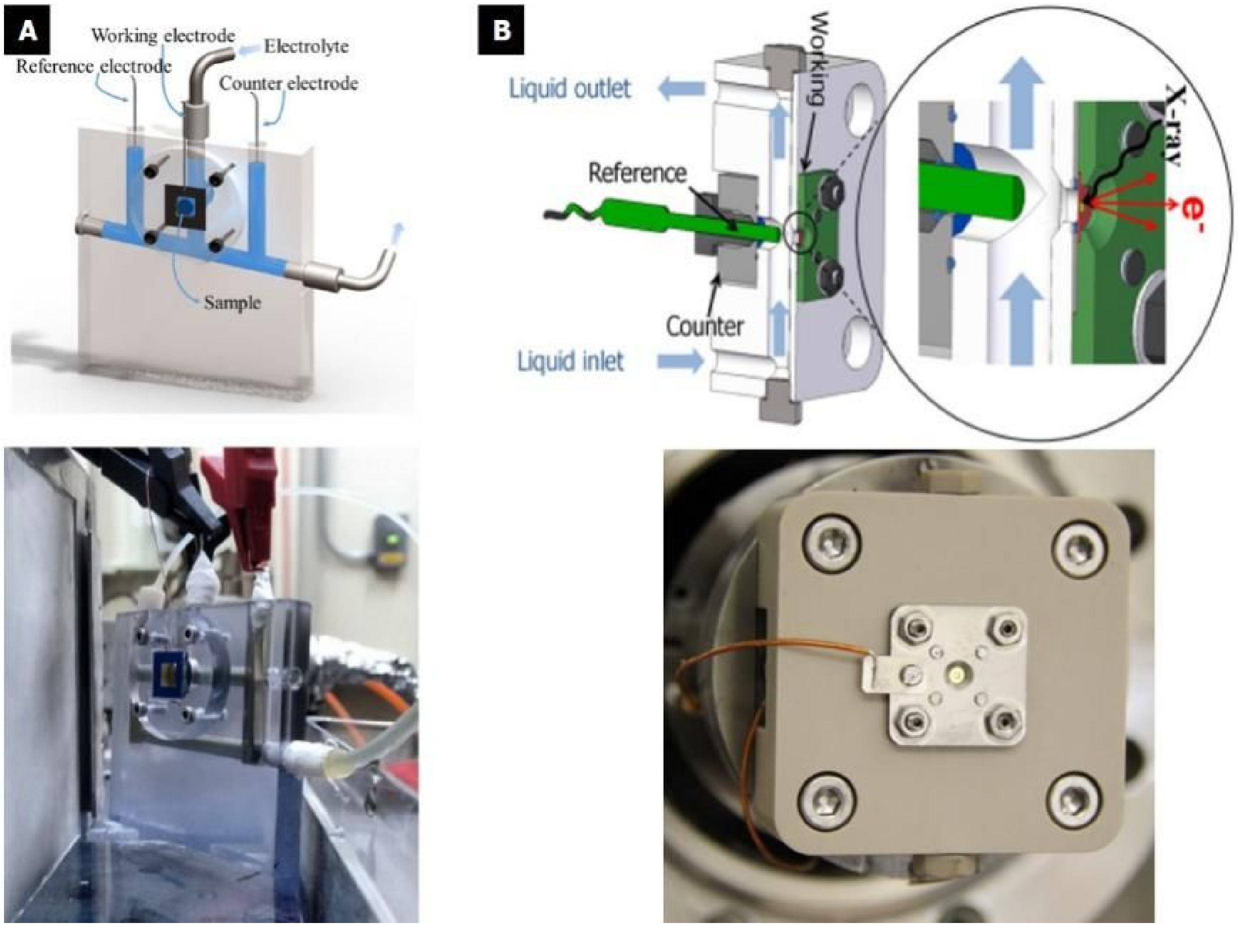

Fig. S2

(A) Sketch and picture of the EC cell used for the hard X-ray measurements in the Ir L_3-edge. (**B**) Sketch and picture of the liquid flow cell used for the PES measurements: The blue arrows indicate the electrolyte flow. The cell has three electrodes: counter, working and reference. The working electrode is transferred directly onto the grid.

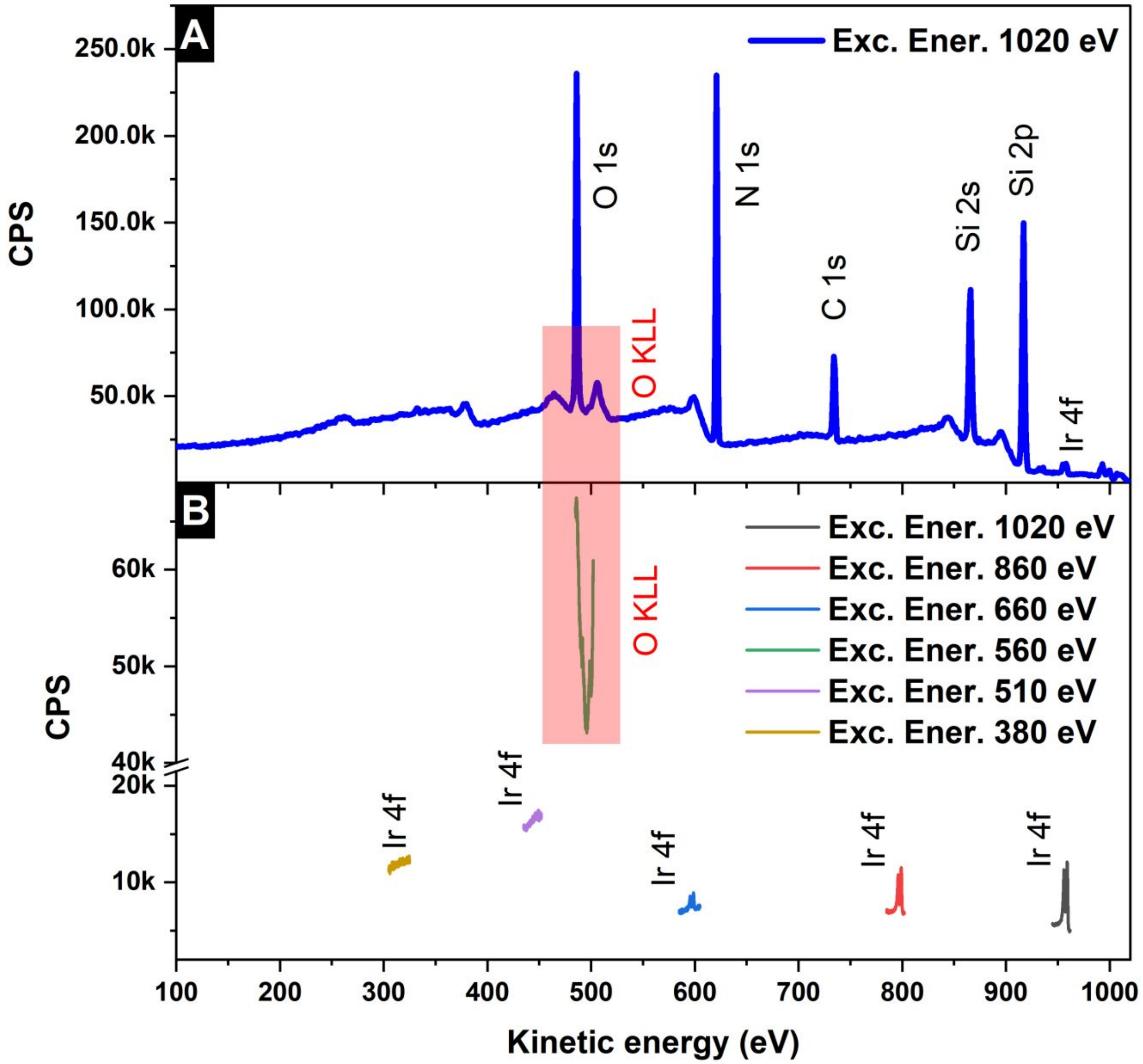

Fig. S3

(A) Survey collected with 1020 eV excitation energy with the Si_3N4/BLG/Ir NPs membrane. The transmission function remains unaltered. The transmission function of the lens and analyser is smooth and not suddenly dropping. (B) Ir 4f spectra collected through the graphene membrane in HV at different KEs. The spectra are not normalized and were plotted as they were collected. Note that in a synchrotron source the excitation energy is tunable.

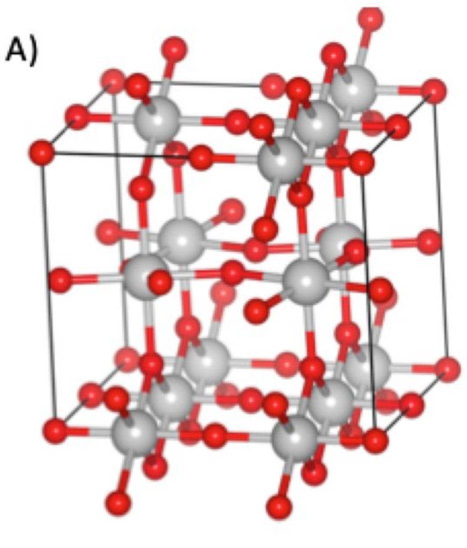

Fig. S4

Structure of bulk Ir_2O_5 **(A)** and bulk IrO_3 **(B)**.

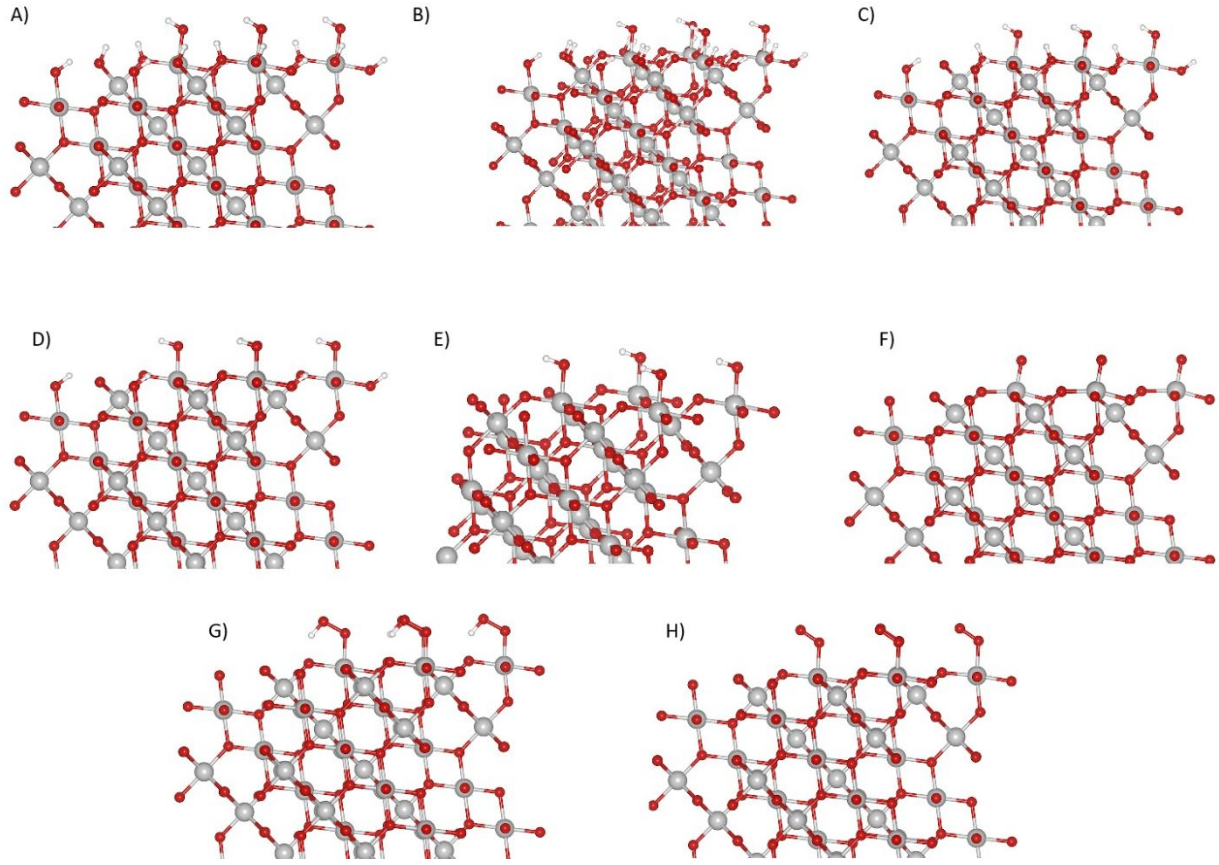

Fig. S5

The surface terminations considered for IrO_2 (111). In **(A)** all surface μ_2-O and μ_1-O are hydroxylated. The μ_2-OH sites are progressively hydroxylated from **(B)** to **(E)**, where in **(E)** the surface has μ_2-O and μ_1-OH. In **(F)** μ_2-O and μ_1-O are present, while further oxidation to **(G)** produces μ_2-O and μ_1-OOH before the most oxidized surface **(H)** is reached, with μ_2-O and μ_1-OO.

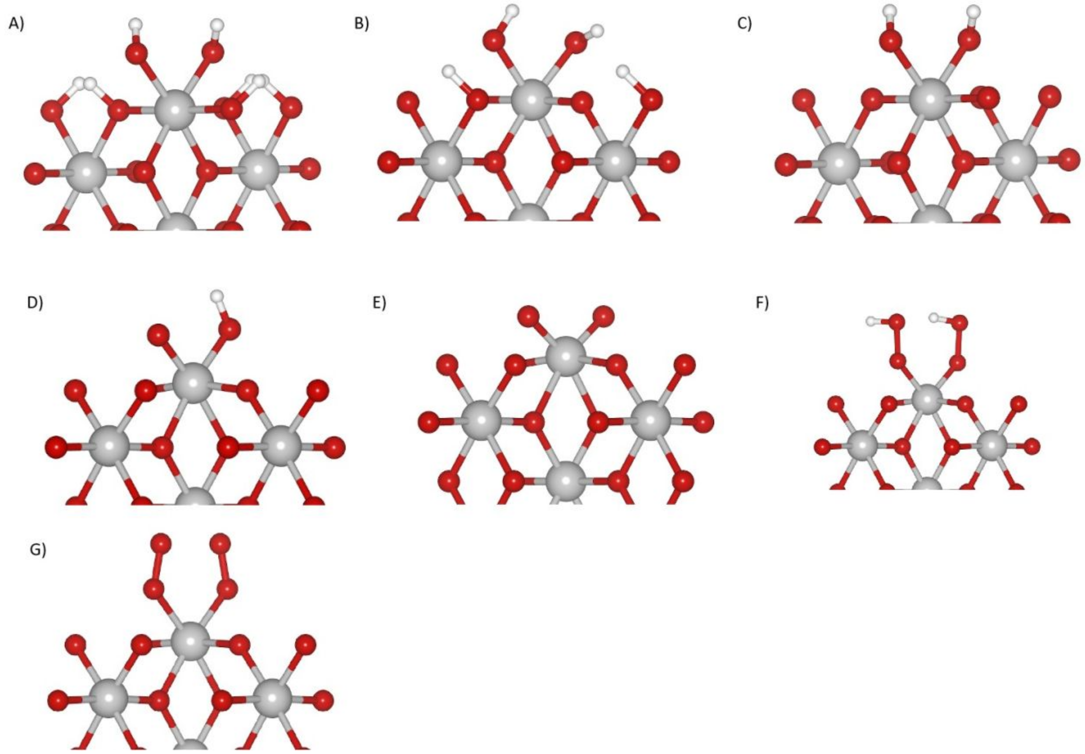

Fig. S6

The surface terminations considered for IrO_2 (001). In **(A)** all surface μ_2-O and μ_1-O are hydroxylated. The μ_2-OH sites are progressively hydroxylated from **(B)** to **(C)**, where in **(C)** the surface has μ_2-O and μ_1-OH. In **(D)** μ_1-OH and μ_1-O are present, while further oxidation to **(E)** produces μ_2-O. Two μ_1-OOH species are shown in **(F)**, and μ_2-O and μ_1-OO in **(G)**.

Table S1: Computed Ir 4f binding energies of iridium bound to μ_1-O(H) for IrO_2 (110) and (111) surfaces with varying degrees of surface oxidation. All μ_2-sites are O.

μ_1-site	(110) Ir 4f / eV	(111) Ir 4f / eV	(001) Ir 4f / eV	(210) Ir 4f
**OH**	61.8	62.5	62.4	61.8
**O**	62.5	63.3	62.8[a], 63.5[b]	62.5
**OOH**	61.9	62.4	62.4	
**OO**	61.6	62.0	61.8	

[a]For the (001) surface the surface Ir is bound to one μ_1-OH and one μ_1-OH.

[b]For the (001) surface the surface Ir is bound to two μ_1-O.

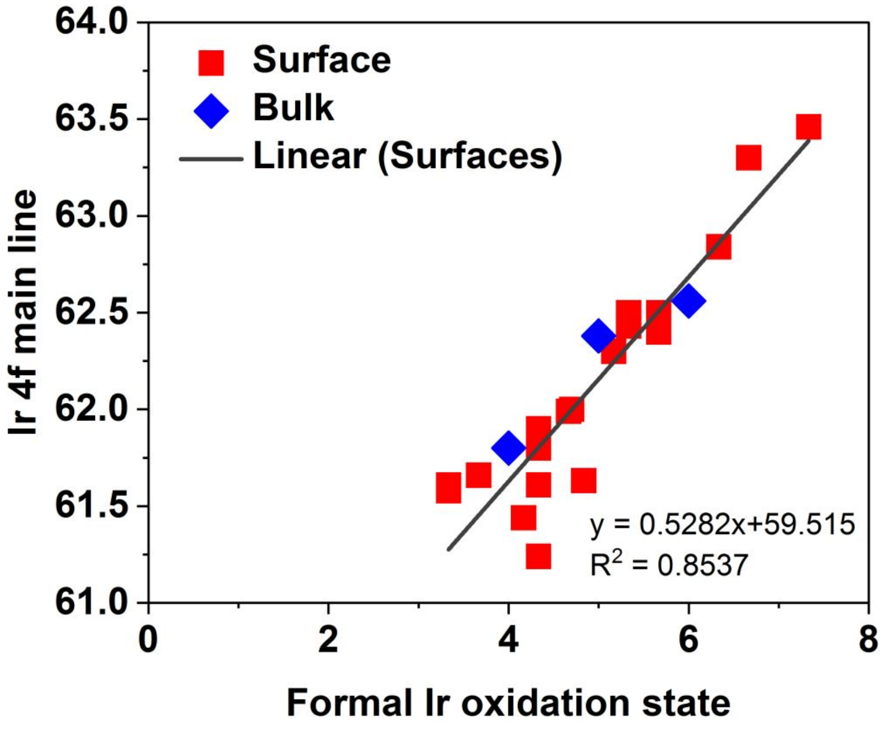

Fig. S7

Correlation between Ir formal oxidation state and Ir 4f shift

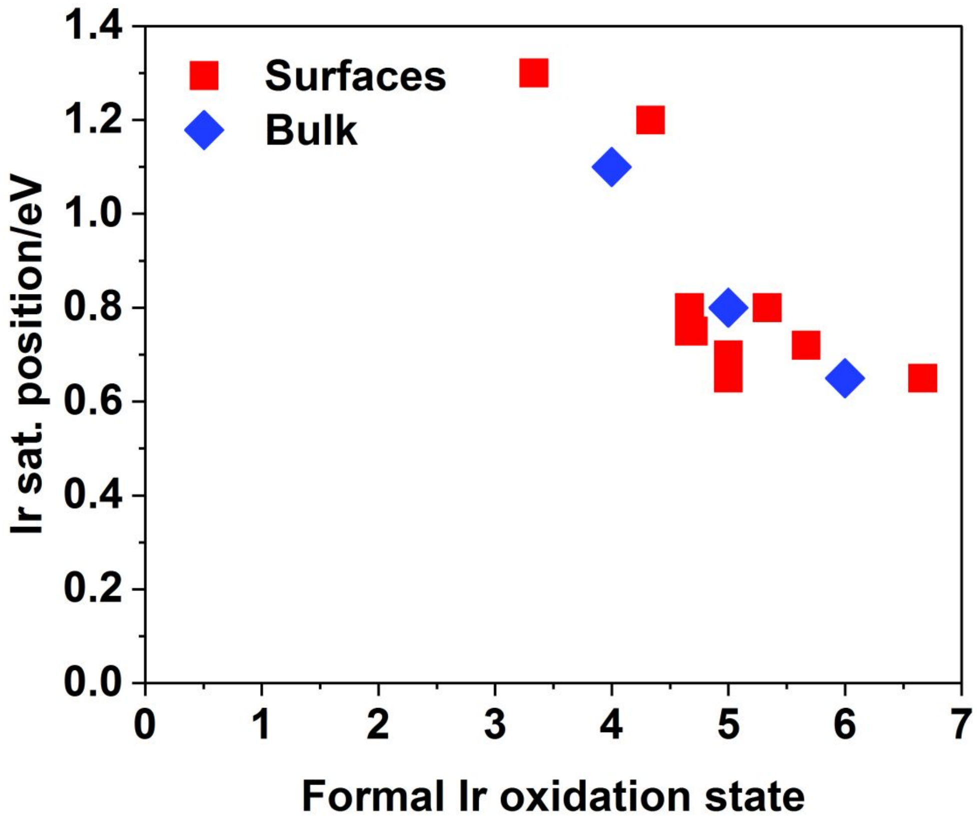

Fig. S8

Correlation between Ir formal oxidation state and satellite position.

Table S2: Parameters used for fitting the *in situ* collected spectra depending on the applied potential using CasaXPS, where DS is referred to an asymmetric Doniach-Sunjic[28] line shape. Meanwhile the GL is referred to a symmetric Gaussian/Lorentzian sum form[29]. The background was subtracted using a Shirley line shape[30].

**Potential**	**Peak**	**Peak possition**	**Peak Shape**	**fwhm**
**+1.6 V vs. Ag/AgCl**	µ1-OH(111)/µ1-O(110)	62.5 eV	DS(0.1;400)	1.1
	$Sat_{\mu1\text{-}OH(111)/\mu1\text{-}O(110)}$ 1	63.3 eV	GL(30)	1.1
	$Sat_{\mu1\text{-}OH(111)/\mu1\text{-}O(110)}$ 2	64.1 eV	GL(30)	1.1
**+1.0 V vs. Ag/AgCl**	µ1-OH(111)/µ1-O(110)	62.5 eV	DS(0.1;400)	1.1
	$Sat_{\mu1\text{-}OH(111)/\mu1\text{-}O(110)}$ 1	63.3 eV	GL(30)	1.1
	$Sat_{\mu1\text{-}OH(111)/\mu1\text{-}O(110)}$ 2	64.1 eV	GL(30)	1.1
	µ1-OH(110)/	61.8 eV	DS(0.1;400)	1.1
	$Sat_{\mu1\text{-}OH(110)}$ 1	62.6 eV	GL(30)	1.1
	$Sat_{\mu1\text{-}OH(110)}$ 2	63.4 eV	GL(30)	1.1
**+0.7 V vs. Ag/AgCl**	Ir^{III}	62.3 eV	GL(30)	1.8
**OCV**	Ir^{III}	62.3 eV	GL(30)	1.8
**HV (reference)**	Ir^{0}	61.2 eV	DS(0.1;400)	1.1

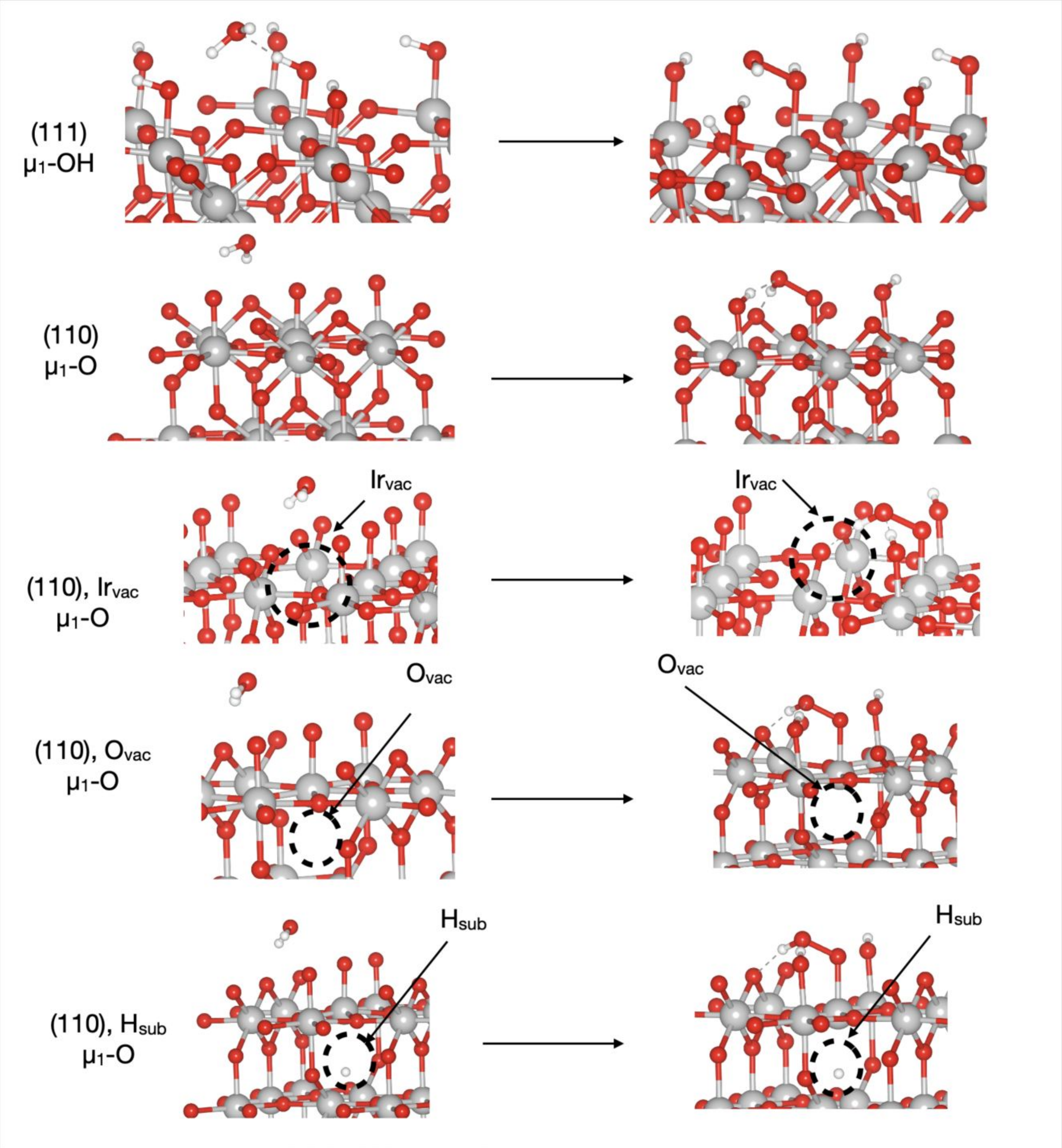

Fig. S9

Ball and stick models of the initial and final states in O-O bond formation on the surfaces used in Figure 7 of the main text. Iridium are shown as grey, oxygen red, and hydrogen white. Each reaction involves forming an O-O bond between μ_1-O(H) and H_2O in a purely chemical step. The μ_1-OH transforms in μ_1-OHOH and the μ_1-O transforms in μ_1-OOH. In both cases the extra H is adsorbed an a different surface oxygen site, a μ_2-O for the (111) and a μ_1-O for the (110) surfaces. The oxidation state of Ir involved in O-O coupling in the (110) surfaces was varied by introducing a surface Ir vacancy, subsurface O vacancy, and subsurface H substitution of O as shown above. Note for the Ir surface vacancy a (2 × 2) supercell was employed.

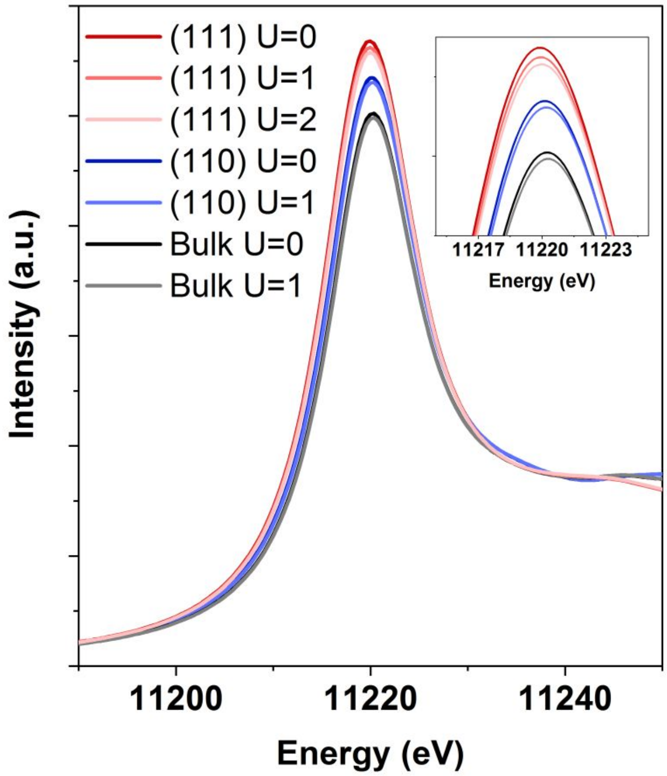

Fig. S10

Ir L_3-edge spectra computed with a Hubbard U of 1 to 2 eV on Ir. The (110) surface is covered in μ_2-O and μ_1-O while the (111) surface has μ_2-O and μ_1-OH (Fig. S6E). These were chosen as the most relevant surfaces since they were assigned from experiment under OER.

References

1. Velasco-Vélez, J. J.; Pfeifer, V.; Hävecker, M.; Wang, R.; Centeno, A.; Zurutuza, A.; Algara-Siller, G.; Stotz, E.; Skorupska, K.; Teschner, D.; Kube, P.; Braeuninger-Weimer, P.; Hofmann, S.; Schlögl, R.; Knop-Gericke, A. Atmospheric pressure X-ray photoelectron spectroscopy apparatus: Bridging the pressure gap. *Rev. Sci. Instr.* **2016**, *87*, 053121.

2. Velasco-Vélez, J. J.; Teschner, D.; Girgsdies, F.; Hävecker, M.; Streibel, V.; Willinger, M. G.; Cao, J.; Lamoth, M.; Frei, E.; Wang, R.; Centeno, A.; Zurutuza, A.; Hofmann, S.; Schlögl, R.; Knop-Gericke, A. The Role of Adsorbed and Subsurface Carbon Species for the Selective Alkyne Hydrogenation Over a Pd-Black Catalyst: An Operando Study of Bulk and Surface. *Top. Cat.* **2018**, *61*, 2052-2061.

3. Velasco-Velez, J. J.; Chuang, C.H.; Han, H.-L.; Martinez-Fernandez, I.; Martinez, C.; Pong, W.-F.; Shen, Y.-R.; Wang, F.; Zhang, Y.; Guo, J.; Salmeron, M. In-situ XAS investigation of the

effect of electrochemical reactions on the structure of graphene in aqueous electrolytes. *J. Electrochem. Soc.*, **2013**, *160*, C445-C450 (2013).

4. Velasco-Velez, J. J.; Wu, C. H.; Sun,Wang, B. Y.; Sun, Y.; Zhang, Y.; Guo, J.-H.; Salmeron, M. Polarized X-ray Absorption Spectroscopy Observation of Electronic and Structural Changes of Chemical Vapor Deposition Graphene in Contact with Water. *J. of Phys. Chem. C* **2014**, 118, 25456-25459.

5. Tuinstra, F.; Koening, J. L. Raman spectrum of graphite. *J. Chem. Phys.* **1970**, *53*, 1126-1130.

6. Reich, S.; Thomse, C.; Maultzsch, J. Carbon nanotubes: basics concepts and physical properties. *Weinheim: Willey-VCH Verlag,* **2008**.

7. Wang, Y.; Alsmeyer, D. C.; McCreery, R. L.; Raman spectroscopy of carbon materials: structural basis of observed spectra. *Chem. Matt.* **1990**, *2*, 557-563.

8. Ismach, A.; Penwell, S.; Schwartzberg, A.; Zheng, Maxwell, Javey, A; Bokor, J.; Zhang, Y. Direct Chemical Vapor Deposition of Graphene on Dielectric Surfaces. *Nano Lett.* 20**10**, *10*, 1542-1548.

9. Velasco-Vélez, J. J.; Jones, T.; Gao, D.; Carbonio, E.; Arrigo, R.; Hsu, C.-J.; Huang, Y.-C.; Dong, C.-L.; Chen, J.-M.; Lee, J.-F.; Strasser, P.; Roldan Cuenya, B.; Schlögl, R.; Knop-Gericke, A.; Chuang, C.-H. The role of the copper oxidation state in the electrocatalytic reduction of CO_2 into valuable hydrocarbons. *ACS Sus. Chem. & Eng.*, **2018**, *7*, 1485-1492.

10. Knop-Gericke, A.; Pfeifer, V.; Velasco-Vélez, J. J.; Jones, T., Arrigo, R.; Hävecker, M.; Schlögl, R. In situ X-ray photoelectron spectroscopy of electrochemically active solid-gas and solid-liquid interfaces. *J. Elec. Spec. Rel. Phenom.* **2017**, *221*, 10-17.

11. Klyushin, A.; Arrigo, R.; Pfeifer, V.; Jones, T:; Velasco Vélez, J. J.; A. Knop-Gericke, Catalyst electronic surface structure under gas and liquid environments. *In Encyclopedia of Interfacial Chemistry, Elsevier* **2018**, 615-631,2018.

12. Giannozzi, P.; Baroni, S.; Bonini, N.; Clandra, M.; Car, R.; Cavazzoni, C.; et al. QUANTUM ESPRESSO: a modular and open-source software project for quantum simulations of materials. *J. Phys.: Condens. Matter* **2009**, *21*, 395502.

13. Dal Corso, A. Pseudopotentials periodic table: From H to Pu. *Comput. Mater. Sci.* **2014**, *95*, 337-350.

14. Ping, Y.; Galli, G.; Goddard III, W. A. Electronic structure of IrO2: the role of the metal d orbitals. *The J. of Phys. Chem. C* **2015**, 119, 11570-11577.

15. Tang, R.; Nie, Y.; Kawasaki, J. K.; Kuo, D. Y.; Petretto, G.; Hautier, G., Rignanese, G.M.; Shen, K.; Suntivich, J. Oxygen evolution reaction electrocatalysis on SrIrO 3 grown using molecular beam epitaxy. *J. of Mat. Chem. A* **2016**, 4, 6831-6836.

16. Cococcioni, M.; De Gironcoli, S. Linear response approach to the calculation of the effective interaction parameters in the LDA+ U method. *Phys. Rev. B* **2005**, 71(3), 035105.

17. Marzari, N.; Vanderbilt, D.; De Vita, A.; Payne, M.C.; Thermal contraction and disordering of the Al (110) surface. *Phys. Rev. Lett.* **1999**, *82*, 3296-3299.

18. Oganov, A.R.; Glass, C. W.; Crystal structure prediction using ab initio evolutionary techniques: Principles and applications. *J. Chem. Phys.* **2006**, *204*, 244704.

19. Jain, A.; Ong, S. P.; Hautier, G.; Chen, W.; Richards, W. D.; Dacek, S.; Cholia, S:; Gunter, D.; Skinner, D.; Ceder, G.; Persson, K. A. Commentary: The materials project: A materials genome approach to accelerating materials innovation. *APL Mater*. **2013**, *1*, 011002.

20. Shirley. E. L. Ti 1s pre-edge features in rutile: a Bethe-Salpeter calculation *J. Electron Spectrosc. Relat. Phenom.* **2004**,, *136*, 77-83.

21. J. Vinson, J.; Rehr, J. J.; Kas, J. J.; Shirley, E. L. Bethe-Salpeter equation calculations of core excitation spectra. *Phys. Rev. B* **2011**, *83*, 115106.
22. Krause, M. O.; Oliver, J. H. Natural widths of atomic K and L levels, K α X-ray lines and several KLL Auger lines. *J. Phys. Chem. Ref. Data* **1979**, *8*, 329-338.
23. E. Pehlke, E.; Scheffler, M. Evidence for site-sensitive screening of core holes at the Si and Ge (001) surface. *Phys. Rev. Lett.* **1993**, *71*, 2338-2341.
24. Hopfield, J. J. Infrared divergences, X-ray edges, and all that. *Comm. Sol. Sta. Phys.* **1969**, *2*, 40-49.
25. Murugappan, K.; Anderson, E: M.; Teschner, D.; Jones, T. E:; Skorupska, K.; Román-Leshkov, Y. Operando NAP-XPS unveils differences in MoO3 and Mo2C during hydrodeoxygenation. *Nat. Cat.* **2018**, *1*, 960–967 (2018).
26. Bunău. O; Calandra, M. Projector augmented wave calculation of x-ray absorption spectra at the $L_{2,3}$ edges. *Phys. Rev. B* **2013**, *87*, 205105.
27. Campbell, J. L.; Papp, T. Widths of the atomic K-N7 levels. *Atomic Data and Nuclear Data Tables* **2001**, *77*, 1–56.
28. Doniach, S.; Sunjic, M. Many-electron singularity in X-ray photoemission and X-ray line spectra from metals. *J. of Phys. C: Sol. Stat. Phys.* **1970**, 3, 285.
29. Shirley, D. A. High-resolution X-ray photoemission spectrum of the valence bands of gold. *Phys. Rev. B* **1972**, 5, 4709.
30. Evans, S. Curve synthesis and optimization procedures for X-ray photoelectron spectroscopy. *Surf. and Interf. Anal.* 1991, 17(2), 85-93.
